# Multi-Target Regulation of Diabetic Nephropathy by Alkaloids: Molecular Targets, Signaling Pathways and Therapeutic Potential

**DOI:** 10.3390/ijms27198938

**Published:** 2026-10-08

**Authors:** Jiying Wang, Qing Li, Wentao Chen, Lingli Zhou, Rongyu Li, Na Zhou, Shifang Luo, Linao Zhang, Xue Wu, Yuhuan Xie, Peixin Guo

**Affiliations:** 1 Yunnan Key Laboratory of Dai and Yi Medicine, Yunnan University of Chinese Medicine, Kunming 650500, China; 15708676760@163.com (J.W.); 15758174180@163.com (Q.L.); a15859361020@163.com (W.C.); zhoulingli0220@163.com (L.Z.); 18053822920@163.com (R.L.); 18487143535@163.com (N.Z.); 18608342484@163.com (S.L.); zla13975@163.com (L.Z.); 15752427670@163.com (X.W.); 2 College of Ethnic Medicine, Yunnan University of Chinese Medicine, Kunming 650500, China; 3 College of Chinese Medicine, Yunnan University of Chinese Medicine, Kunming 650500, China; 4 College of Basic Medical Sciences, Yunnan University of Chinese Medicine, Kunming 650500, China

**Keywords:** alkaloids, diabetic nephropathy, molecular mechanisms, therapeutic prospects

## Abstract

In the context of the global diabetes epidemic, diabetic nephropathy (DN)—a condition directly associated with diabetes—has become the primary cause of end-stage renal disease, significantly impacting patients’ quality of life and placing substantial demands on public healthcare resources. The pathogenesis of DN is characterized by metabolic disturbances induced by high glucose levels, hemodynamic abnormalities, and the synergistic amplification of various stress signals. These factors collectively contribute to the progressive loss of nephrons and irreversible renal dysfunction. Although current standard treatments can partially slow disease progression, they significantly fail to meet clinical needs. Therefore, there is an urgent necessity to identify multi-target intervention strategies that utilize novel mechanisms of action. Alkaloids, a class of natural nitrogen-containing compounds known for their significant structural diversity, have recently demonstrated multiple renoprotective effects in experimental models of DN. This review is based on a search of the PubMed, Web of Science and CNKI databases. The keywords alkaloids, diabetic nephropathy, diabetic nephropathies, diabetic kidney disease, inflammation, oxidative stress, fibrosis, autophagy and apoptosis were used to search for original research articles in both Chinese and English, published between 2010 and 2026, which investigated the mechanisms by which alkaloid compounds exert their anti-DN effects. Focusing on anti-inflammatory effects, anti-oxidative stress, anti-renal fibrosis, autophagy modulation, apoptosis regulation and ferroptosis inhibition, the review then summarized the relevant literature by systematizing the experimental models, intervention protocols, dosages and key results. This provided a new theoretical perspective and experimental basis for the clinical translation and new drug development of alkaloid compounds for the treatment of DN. The review provides a new theoretical perspective and an experimental basis for the clinical translation of, and new drug development based on, alkaloid compounds for the treatment of DN.

## 1. Introduction

Diabetic nephropathy (DN) is one of the most prevalent chronic microvascular complications associated with diabetes mellitus (DM) and serves as a primary contributor to end-stage renal disease (ESRD) [1]. Epidemiological studies indicate that the global prevalence of DM currently stands at 521 million. As the incidence of DM continues to rise, the number of individuals affected by DN is also increasing annually. It is projected that by 2050, the global population of DM patients will reach 1.31 billion, with an estimated 30% to 40% of these individuals likely to develop DN [2,3]. The pathological features of DN primarily include thickening of the glomerular basement membrane, deposition of extracellular matrix, interstitial fibrosis, the formation of nodular glomerulosclerosis, and a reduction in the number of endothelial cells. Clinically, these features are characterized by persistent albuminuria and a progressive decline in glomerular filtration rate [4]. The progressive development of glomerulosclerosis and tubulointerstitial fibrosis leads to a gradual decline in renal function, ultimately resulting in end-stage renal disease (ESRD). Patients with ESRD often require renal replacement therapy to sustain life [5]. This trend poses a significant threat not only to patients’ quality of life but also to healthcare systems worldwide.

The pathogenesis of DN is complex, involving the abnormal activation of various signaling pathways linked to oxidative stress, inflammation, and fibrosis [6]. Currently, the treatment for DN primarily consists of symptomatic therapies aimed at controlling hyperglycemia, dyslipidemia, and hypertension [7]. Currently, angiotensin receptor blockers (ARBs), angiotensin-converting enzyme inhibitors (ACEIs) [8], sodium-glucose cotransporter 2 (SGLT2) inhibitors [9,10], and non-steroidal mineralocorticoid receptor antagonists (NS-MRAs) [11] are in clinical use. However, these agents demonstrate limited efficacy in slowing disease progression, resulting in many patients still advancing to end-stage renal disease (ESRD) [12]. Consequently, it is imperative to thoroughly elucidate the molecular mechanisms underlying the onset and progression of DN and to develop novel therapeutic agents based on these insights, as this has become an urgent priority.

Although strict glycemic control is the fundamental strategy for delaying the onset of DN, the phenomenon of metabolic memory suggests that prior exposure to high glucose levels can induce epigenetic reprogramming, mitochondrial dysfunction and chronic inflammation [13]. Even after blood glucose levels return to normal, these changes may persist in the long term and continue to cause kidney damage to progress. Glucose-lowering interventions alone cannot eliminate residual risk of DN progression completely. Once microalbuminuria develops, a pathological feedback loop can form within the kidney that is relatively independent of systemic blood glucose levels. This involves excessive activation of the intrarenal renin–angiotensin system (RAS), podocyte damage and loss, and abnormal mesangial matrix accumulation. This loop drives the continued worsening of the disease [14]. Therefore, glycemic control aimed at addressing the underlying cause, as well as the prevention and interruption of downstream renal pathological cascades, is significant and both approaches hold clinical importance. Clinical trials that have used markers of kidney damage as exploratory endpoints, independent of the effects of glycemic control, have confirmed that certain medications can exert direct renoprotective effects [15].

Alkaloids are a class of nitrogen-containing basic organic compounds that are widely found in nature and exhibit various pharmacological activities, including antitumor, anti-inflammatory, and hypoglycemic effects [16]. Due to their diverse chemical structures and broad spectrum of biological activity, alkaloids demonstrate multi-pathway, multi-target, and multilevel renal protective effects, providing a unique advantage in addressing the complex pathological processes associated with diabetic nephropathy [17]. To date, various alkaloids, including pyridine derivatives, isoquinoline derivatives, pyrazine derivatives, organic amines, and terpenoid alkaloids, have been shown to alleviate or slow the progression of diabetic nephropathy.

To evaluate the therapeutic potential of alkaloid compounds in DN, we conducted a comprehensive search in the PubMed, Web of Science, and CNKI databases. We utilized keywords such as “alkaloid compounds,” “diabetic nephropathy,” “diabetic nephropathies,” “diabetic kidney disease,” “inflammation,” “oxidative stress,” “fibrosis,” “autophagy,” “apoptosis,” and “ferroptosis” to identify relevant articles published between 2010 and 2026. This article classifies and discusses alkaloids based on their molecular mechanisms, including anti-inflammatory, antioxidant, antifibrotic, autophagy-modulating, apoptosis-modulating, and anti-ferroptotic effects. Additionally, it analyzes the structure–activity relationships of each compound. Furthermore, this study summarizes the current status of clinical translation, identifies existing bottlenecks, and proposes strategies to overcome these challenges. This provides a foundation for translating alkaloid compounds from preclinical research into therapeutic drugs for the treatment of DN and offers a novel perspective on the design of multi-target drugs derived from natural products.

## 2. Literature Search and Screening

This review conducted a comprehensive search of the PubMed, Web of Science, and CNKI databases for articles published between 2010 and 2026. The CNKI database utilized Chinese language search terms. The scope of the search encompassed titles, keywords, and abstracts, with searches employing the Chinese equivalents of “diabetic nephropathy” and “alkaloids.”

PubMed Search Query: (“alkaloids” [MeSH Terms] OR “alkaloid” [Title/Abstract]) AND (“diabetic nephropathies” [MeSH Terms] OR “diabetic nephropathy” [Title/Abstract] OR “diabetic kidney disease” [Title/Abstract]).

Web of Science Search Query: TS = (alkaloids OR alkaloid) AND TS = (diabetic nephropathies OR diabetic nephropathy OR diabetic kidney disease).

CNKI Search Query: (Title, Keywords, and Abstract: “diabetic nephropathy”) AND (Title, Keywords, and Abstract: “alkaloids”).

Following an initial screening of titles and abstracts, we excluded the literature that was clearly irrelevant to the topic, not written in Chinese or English, or which was duplicated or retracted. Additionally, we excluded the literature for which the full text was unavailable. Ultimately, we selected the literature pertinent to the study of the mechanisms by which alkaloid compounds combat DN, with a particular focus on those closely associated with anti-inflammatory, antioxidant, anti-fibrotic, autophagy-regulating, apoptosis-regulating, and anti-ferroptotic effects. The experimental models, intervention protocols, dosages, and key results of these studies were subsequently organized and summarized.

## 3. Current Status of Clinical Translation of Alkaloid Therapy for Diabetic Nephropathy

Based on published clinical research evidence, berberine is currently undergoing clinical trials as a dietary supplement for adjunctive intervention in blood glucose regulation and metabolism. Multiple randomized controlled trials have confirmed its nephroprotective effects. In a randomized controlled clinical trial involving 69 patients with hypertension and type 2 diabetes, participants were randomly assigned to either the control group (33 patients) or the treatment group (36 patients). The control group received standard antihypertensive and antidiabetic therapy, while the treatment group received berberine (0.1 g per dose, three times daily) in conjunction with standard therapy for a duration of 24 consecutive months. Efficacy was assessed using indicators such as the urinary albumin-to-creatinine ratio (UACR), urinary kidney injury molecule-1 (KIM-1), and osteopontin. The results indicated that blood pressure and blood glucose levels were well controlled in both groups. However, the UACR increased in the control group compared to baseline measurements. Conversely, patients in the berberine treatment group exhibited a significant reduction in UACR compared to the control group, with values decreasing from 63.33 μg/mg to 53.37 μg/mg, while the control group experienced an increase from 65.18 μg/mg to 87.04 μg/mg. Furthermore, improvements in early kidney injury markers, such as urinary KIM-1 and osteopontin, as well as renal hemodynamics, were more pronounced in the berberine treatment group than in the control group [18]. In addition, a study was conducted involving 114 patients with type 2 diabetes. Participants were randomly assigned to either a control group or an intervention group, with 57 patients in each group. The control group received standard antidiabetic treatment, while the intervention group received berberine (0.4 g per dose, three times daily) in conjunction with standard antidiabetic treatment for six consecutive months. UACR levels decreased significantly in both groups compared to pretreatment levels, with a greater reduction observed in the intervention group (from 97 mg/g to 68 mg/g in the control group and from 103 mg/g to 47 mg/g in the intervention group). Concurrently, serum cystatin C levels also showed a more pronounced decrease. Furthermore, multiple linear regression analysis confirmed that berberine administration was an independent factor contributing to the reduction in UACR and cystatin C levels, indicating a favorable treatment outcome [19].

Several Phase II–IV trials evaluating the efficacy of berberine in patients with diabetes have been registered on ClinicalTrials.gov (see Table 1A,B). Among these, NCT05749874 stands out as the largest ongoing phase IV trial, with 2024 participants currently enrolled and an expected conclusion in December 2026. The exploratory endpoints of this study include changes in UACR, which is a crucial indicator for the early diagnosis and monitoring of DN progression. Positive outcomes from this research would provide compelling indirect evidence that berberine may slow the progression of DN.

Sinomenine is the primary active ingredient in the traditional Chinese medicinal herb Sinomenium acutum (Thunb.) Rehd. et Wils., which has substantial clinical evidence supporting its efficacy in treating DN. In a randomized controlled trial involving 64 patients with early-stage DN, Zeng Fanxue divided the participants into two groups: a control group (32 patients) receiving standard treatment—including glucose-lowering, blood pressure-lowering, and lipid-lowering therapies, as well as a low-salt, low-fat diet—and a treatment group (32 patients) that received the same standard treatment supplemented with Zheng Qingfeng Tongning sustained-release tablets (120 mg, twice daily). After 8 weeks of treatment, the treatment group exhibited significantly greater improvements in serum inflammatory markers, including the UACR, 24 h urine protein quantification, tumor necrosis factor-α (TNF-α), interleukin-6 (IL-6), and high-sensitivity C-reactive protein (hs-CRP), compared to the control group (*p* < 0.01). Additionally, the overall clinical response rate in the treatment group was 81.25%, which was significantly higher than the 68.75% observed in the control group (*p* < 0.05) [20]. None of the studies included in this review reported any serious adverse reactions. A small number of patients experienced mild rashes or gastrointestinal discomfort, all of which resolved spontaneously. These findings suggest that the sinomenine preparation, Zheng Qingfeng Tongning sustained-release tablets, demonstrates both good efficacy and safety as an adjunctive treatment for DN.

The primary active alkaloid in the traditional Chinese medicine *Ligusticum sinense* (*Chuanxiong*) is tetramethylpyrazine, which has demonstrated significant efficacy in the adjunctive treatment of DN. Zou Junfeng further corroborated these findings in a randomized controlled trial involving 128 patients diagnosed with DN. The patients were randomly assigned to either a control group or an observation group, each consisting of 64 patients. The control group received standard treatment, which included hypoglycemic and antihypertensive therapies, along with a low-protein diet. Conversely, the observation group received the same standard treatment, supplemented with a Chuanxiongqin injection (80 mg per dose), dissolved in a 5% glucose solution and administered via intravenous infusion. After four weeks of continuous treatment, urinary levels of N-acetyl-β-D-glucosaminidase (NAG), retinol-binding protein (RBP), α1-microglobulin (α1-MG), β2-microglobulin (β2-MG), and collagen type IV (Col-IV) were significantly lower in the observation group compared to the control group (all *p* < 0.05). Additionally, serum levels of hyaluronic acid (HA), laminin (LN), procollagen type III (PC-III), and collagen type III (Col-III) were also significantly reduced. Blood urea nitrogen (BUN) levels were notably lower in the observation group than in the control group (*p* < 0.05). Moreover, twenty-four-hour urinary protein excretion was decreased in the observation group, while plasma albumin levels and the rate of tubular reabsorption of albumin were elevated (*p* < 0.05) [21]. These results suggest that Chuanxiongqin injection may delay the progression of DN by protecting renal tubules, inhibiting renal interstitial fibrosis, and improving microcirculation. Therefore, it holds potential as an adjunctive treatment for DN.

Ramulus Mori (Sangzhi) Alkaloids (SZ-As) are alkaloid compounds derived from Mulberry Twig. In 2020, they received approval for marketing in China as a Category 1.2 innovative traditional Chinese medicine for the treatment of type 2 diabetes. A clinical study involving 90 patients with DN demonstrated that the control group (*n* = 45) received standard therapy, which included acarbose (50 mg three times daily) in combination with a sartan (80 mg once daily). In contrast, the treatment group (*n* = 45) received the same regimen with the addition of SZ-As (100 mg three times daily). After 12 weeks of treatment, the treatment group exhibited significantly greater improvements compared to the control group in renal function indicators (creatinine (Cr), BUN, estimated glomerular filtration rate (eGFR)), glycemic indicators (fasting plasma glucose (FPG), 2h-PG, HbA1c), and lipid profile indicators (TC, TG, LDL-C) (*p* < 0.05) [22]. Based on existing clinical evidence, the ‘Expert Consensus on Screening and Prevention of Diabetic Microvascular Disease in Primary Care (2024, China)’ recommends SZ-As (one to two tablets per dose, three times daily) for the treatment of DN [23]. The China Clinical Trial Registry has registered several clinical studies investigating the use of SZ-As in the treatment of DN (registration numbers: ChiCTR2600127423 and ChiCTR2500115748). These studies aim to further evaluate the renoprotective effects of SZ-As and elucidate their specific mechanisms in patients with DN. The results of these studies are highly anticipated.

In summary, berberine, sinomenine, tetramethylpyrazine, and SZ-As have all demonstrated varying degrees of nephroprotective effects in the clinical treatment of DN. Among these, berberine has the most extensive data from registered clinical trials, including the world’s largest phase IV study (NCT05749874). Multiple randomized controlled trials have independently validated berberine’s renoprotective effects, independent of its hypoglycemic action. However, the aforementioned clinical studies have several limitations. Firstly, the sample sizes of the included studies were generally small, and most were single-center studies, resulting in inconsistent study quality. Secondly, the follow-up periods in most studies were relatively short (8–12 weeks), and long-term efficacy and safety data are lacking. Thirdly, clinical studies of these alkaloids have primarily been conducted in Chinese populations, and their efficacy and safety in different ethnic groups remain to be verified. Future research should involve rigorously designed, large-sample, multicenter randomized controlled trials with sufficient follow-up periods. These trials should use standardized renal endpoints (such as the incidence of ESRD and the annual decline in eGFR) as primary outcome measures. This approach will help clarify the role and significance of these alkaloids in the treatment of DN.

## 4. Mechanisms of Action of Alkaloid Compounds in the Treatment of Diabetic Nephropathy

**Figure 1:**
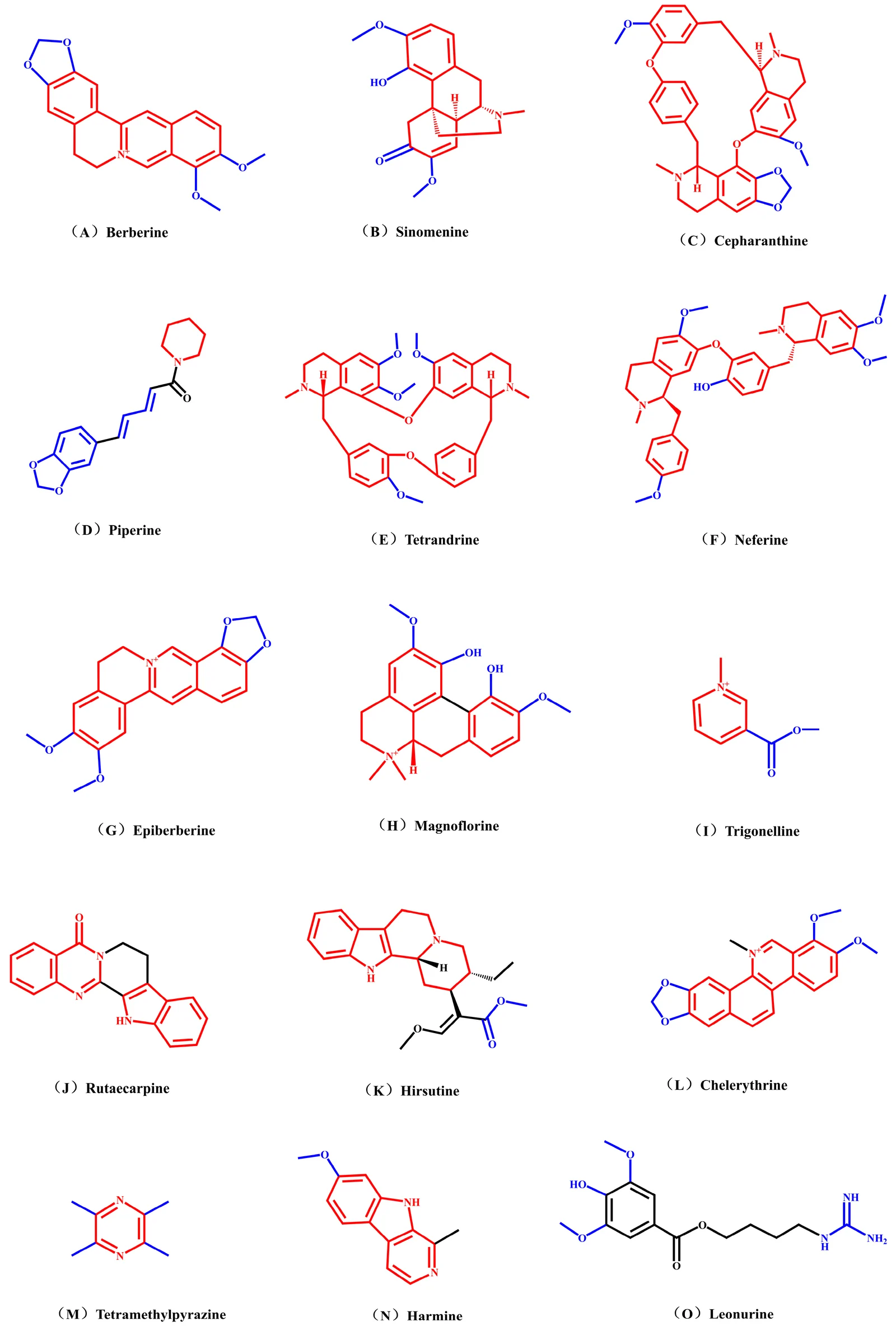
Chemical structures of alkaloids. (**A**) berberine; (**B**) sinomenine; (**C**) cepharanthine; (**D**) piperine; (**E**) tetrandrine; (**F**) neferine; (**G**) epiberberine; (**H**) magnoflorine; (**I**) trigonelline; (**J**) rutaecarpine; (**K**) hirsutine; (**L**) chelerythrine; (**M**) tetramethylpyrazine; (**N**) harmine; (**O**) leonurine Red represents the parent nucleus; blue represents the substituent. (Drawn using ChemDraw 22.0).

Studies have demonstrated that alkaloid compounds can influence the pathological processes of DN through multi-target mechanisms. These mechanisms primarily include anti-inflammatory effects, antioxidant properties, anti-renal fibrosis effects, regulation of autophagy, modulation of apoptosis, and prevention of ferroptosis. Such mechanisms provide a significant theoretical foundation for developing multi-target treatment strategies for DN utilizing alkaloid compounds. It is noteworthy that the protective effects of alkaloids against DN operate through two primary mechanisms. Firstly, alkaloids can directly target the kidneys to intervene in pathological processes, including inflammation, oxidative stress, fibrosis, and programmed cell death. Secondly, certain alkaloids may mitigate the risk of DN by regulating systemic glucose and lipid metabolism, modulating the gut microbiota, and safeguarding the vascular endothelium [24,25,26]. Table 2 summarizes the mechanisms of action of 15 representative alkaloid compounds against DN, with their chemical structures illustrated in Figure 1. Detailed information regarding the plant sources, traditional uses, and modern pharmacological activities can be found in Supplementary Table S1 (Excel file). The subsequent section will concentrate on the regulatory effects of alkaloids on various pathogenic pathways within kidney tissues.

### 4.1. Anti-Inflammatory Response

Chronic low-grade inflammation is a hallmark of DN throughout its progression [44]. Elevated glucose levels, advanced glycation end products (AGEs), and reactive oxygen species (ROS) activate the innate immune system, resulting in the infiltration of macrophages and T lymphocytes into the kidneys. This infiltration prompts intrinsic renal cells, such as mesangial cells and renal tubular epithelial cells, to secrete substantial amounts of TNF-α, interleukin-1β (IL-1β), IL-6, monocyte chemoattractant protein-1 (MCP-1), and other inflammatory mediators and chemokines [45]. Through autocrine and paracrine mechanisms, these factors perpetually activate inflammatory signaling pathways, including the TLR4/NF-κB signaling pathway and the NLRP3 inflammasome pathway. These pathways not only directly damage renal cells but also promote cellular phenotypic transformation and extracellular matrix synthesis, thereby fostering conditions that are conducive to renal fibrosis [46]. Consequently, targeting the regulation of inflammatory responses has emerged as a critical strategy for intervening in DN.

#### 4.1.1. The NF-κB Signaling Pathway

**Figure 2:**
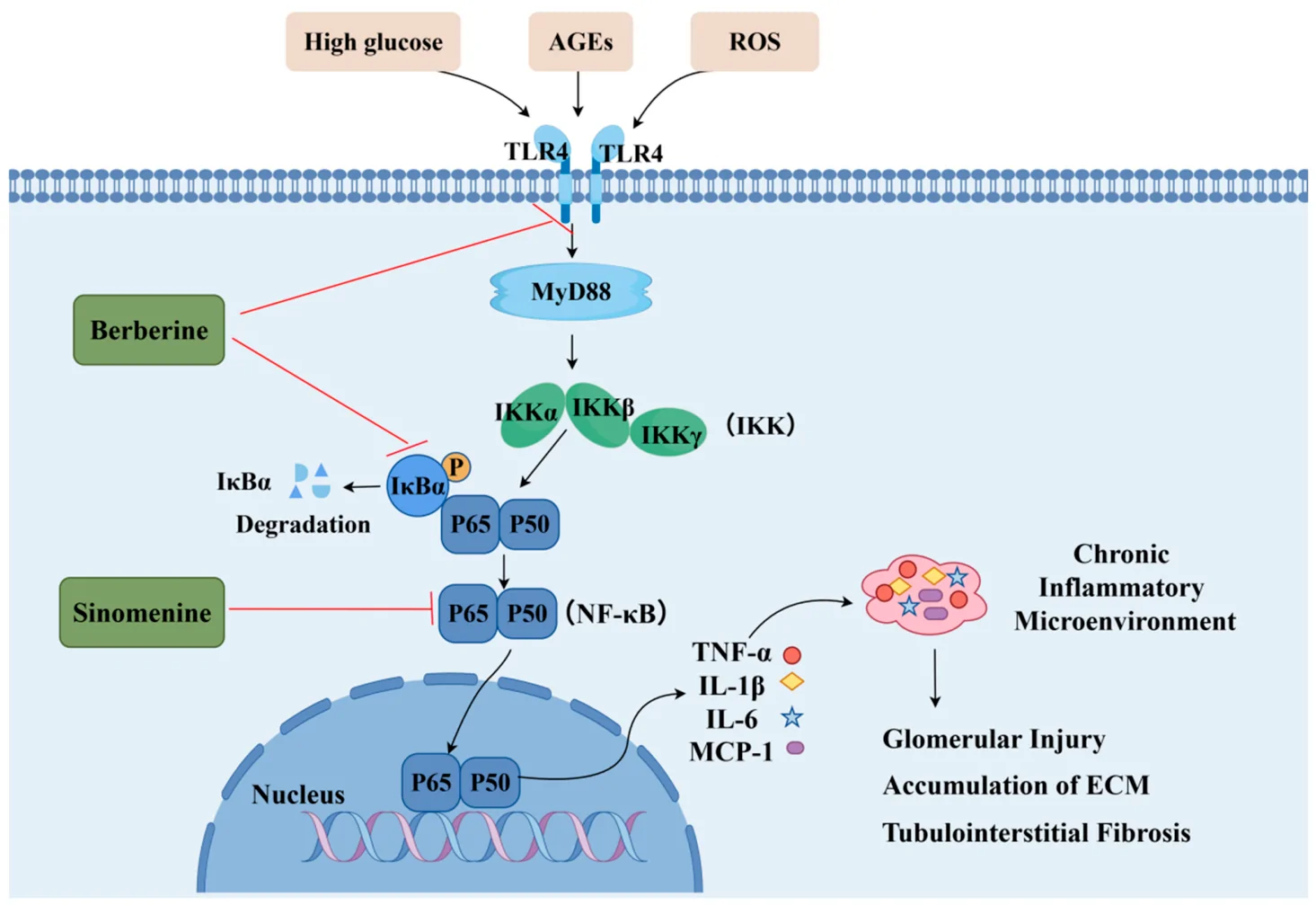
Alkaloids inhibit the activation of the NF-κB signaling pathway. Arrows (→) indicate activation or signal flow, while blunt-ended lines (⊣) indicate inhibition. Chemical structures of the compounds involved in this study, such as (A) berberine and (B) sinomenine, are depicted in Figure 1. Created with Figdraw (by https://www.figdraw.com).

The NF-κB signaling pathway plays a critical role in regulating the inflammatory response. In the context of DN, pathological factors such as high glucose levels, AGEs, and ROS can activate TLR4. Upon activation, TLR4 utilizes the adaptor protein MyD88 to activate the NF-κB inhibitor kinase complex (IKKα/IKKβ/IKKγ, IKK). Subsequently, IKK phosphorylates the serine residues at the regulatory sites of the IκBα subunit, leading to the dissociation of IκBα and the release of the NF-κB dimer formed by p65 and p50. This p65-p50 dimer then translocates to the nucleus, where it initiates the transcription of various pro-inflammatory cytokine genes. Consequently, there is a substantial release of TNF-α, IL-1β, IL-6, and MCP-1. This process creates a local chronic inflammatory microenvironment in the kidney, which accelerates glomerular injury, extracellular matrix (ECM) accumulation, and the progression of tubulointerstitial fibrosis [47,48] (see Figure 2).

Berberine is a quaternary ammonium isoquinoline alkaloid derived from the dried bark of the *Phellodendron amurense* Rupr., belonging to the genus Phellodendron in the family Rutaceae. It demonstrates a range of pharmacological activities, including hypoglycemic, hypolipidemic, antibacterial, and anti-inflammatory effects [49]. Its key features include a rigid planar tetrahedral framework and a permanently charged quaternary ammonium nitrogen center. The quaternary ammonium nitrogen facilitates the formation of ionic bonds with acidic amino acid residues in the TLR4/MD-2 complex, thereby blocking receptor dimerization and effectively inhibiting the activation of key downstream inflammatory signaling pathways, such as NF-κB [50,51]. A study utilizing a Sprague-Dawley (SD) rat model of DN, induced by a single intraperitoneal injection of streptozotocin (STZ) at a dose of 65 mg/kg, demonstrated that intragastric administration of berberine at doses of 50, 100, and 200 mg/kg once daily over a period of eight consecutive weeks effectively reduced fasting blood glucose levels. The findings indicated that berberine led to a dose-dependent decrease in fasting blood glucose, kidney-to-body weight ratio, 24 h urinary protein excretion, serum creatinine (Scr) levels, and blood urea nitrogen (BUN) levels in the DN rats. Furthermore, berberine inhibited the activation of the TLR4/NF-κB signaling pathway and suppressed TLR4 expression, along with the phosphorylation of p-IκBα and p-p65 in the renal tissue of the DN rats. Additionally, berberine reduced the levels of IL-1β, IL-6, and MCP-1. In an in vitro study, mouse podocytes were exposed to 30 mM glucose for 24 h, followed by treatment with 10, 30, and 90 μM berberine for an additional 24 h. The results revealed that berberine significantly decreased the expression of IL-1β, IL-6, and MCP-1 and inhibited the activation of the TLR4/NF-κB pathway [27]. In a separate in vitro study, mesangial cells (MCs) were isolated from the glomeruli of SD rats. The cells were pretreated with berberine at concentrations of 10, 30, and 90 μM for 24 h. Subsequently, an MCs inflammation model was induced using 10 nM lipopolysaccharide (LPS). The results indicated that 30 min after the induction of the MCs inflammation model with LPS, berberine effectively inhibited the activation of the NF-κB signaling pathway by preventing the degradation of IκBα and the nuclear translocation of NF-κB p65. This intervention significantly reduced the expression of NF-κB p65 protein in the MCs nuclei while concurrently upregulating cytoplasmic p65 expression and IκBα protein levels. Twelve hours post-induction of the inflammatory model in MCs, berberine downregulated ICAM-1 and iNOS protein expression in a concentration-dependent manner [28]. These findings suggest that berberine inhibits the early activation of the NF-κB signaling pathway, subsequently blocking the expression of inflammatory mediators and adhesion molecules at the transcriptional level. This effectively ameliorates the local inflammatory state in the kidneys of patients with DN and delays the pathological progression of the disease.

Sinomenine is a morphinan-type isoquinoline alkaloid extracted from the rhizomes of *Sinomenium acutum* (Thunb.) Rehd. et Wils., a plant belonging to the Sinomenium genus in the Menispermaceae family. It is most commonly utilized in its hydrochloride form and exhibits anti-inflammatory, immunosuppressive, cardioprotective, and neuroprotective effects [52]. Its molecular structure is characterized by a fused tetracyclic skeleton, a phenolic hydroxyl group at the C-4 position, a methoxy group at the C-3 and C-7 positions, and a tertiary nitrogen atom at the N-17 position. The C-4 position is particularly crucial for modulating anti-inflammatory activity; introducing a halogen atom or an appropriately sized ester side chain can enhance the inhibition of NF-κB activity. Furthermore, the methoxy group at the C-3 and C-7 positions is significant for anti-inflammatory activity, with the introduction of hydrophobic groups leading to a marked enhancement of this effect. Additionally, incorporating heterocycles, such as triazoles, can improve binding to the NF-κB p50 protein, thereby significantly increasing its anti-inflammatory activity [53]. A study utilizing 12-week-old male db/db mice as a model for DN demonstrated that intragastric administration of sinomenine at a dosage of 62.4 mg/kg once daily for eight consecutive weeks significantly enhanced the overall health and renal function of the mice. This treatment also resulted in a reduction of 24 h urinary microalbumin (mALB), as well as serum levels of Cys-C, Scr, and BUN. Furthermore, it alleviated pathological and ultrastructural damage, which included tubular and interstitial dilatation, glycogen deposition, podocyte fusion, and thickening of the basement membrane. Concurrently, sinomenine significantly downregulated the expression of cGAS and STING by inhibiting the cGAS/STING/NF-κB signaling pathway. This inhibition suppressed the phosphorylation of downstream signaling molecules, TBK1 and IRF3, thereby blocking the nuclear translocation and transcriptional activity of NF-κB. Ultimately, this led to a reduction in the secretion of pro-inflammatory factors such as IL-1β, IL-6, and TNF-α, mitigating renal inflammatory damage in DN and enhancing renal function. Thus, sinomenine exhibited a nephroprotective effect [29]. In addition, a study established a rat model of DN through a single intraperitoneal injection of 60 mg/kg STZ. Subsequently, the rats were administered sinomenine at doses of 20 or 40 mg/kg via oral gavage once daily for six consecutive weeks. In vitro, human glomerular endothelial cells (HrGECs) were treated with 30 mM high glucose and either 50 or 100 μM sinomenine for 24 h. The results indicated that sinomenine significantly reduced the mRNA and protein levels of IL-1β and IL-18 in the kidney tissue of DN rats and in high-glucose-induced HrGECs, thereby alleviating inflammatory cell infiltration in kidney tissue [30].

#### 4.1.2. The NLRP3 Inflammasome Signaling Pathway

**Figure 3:**
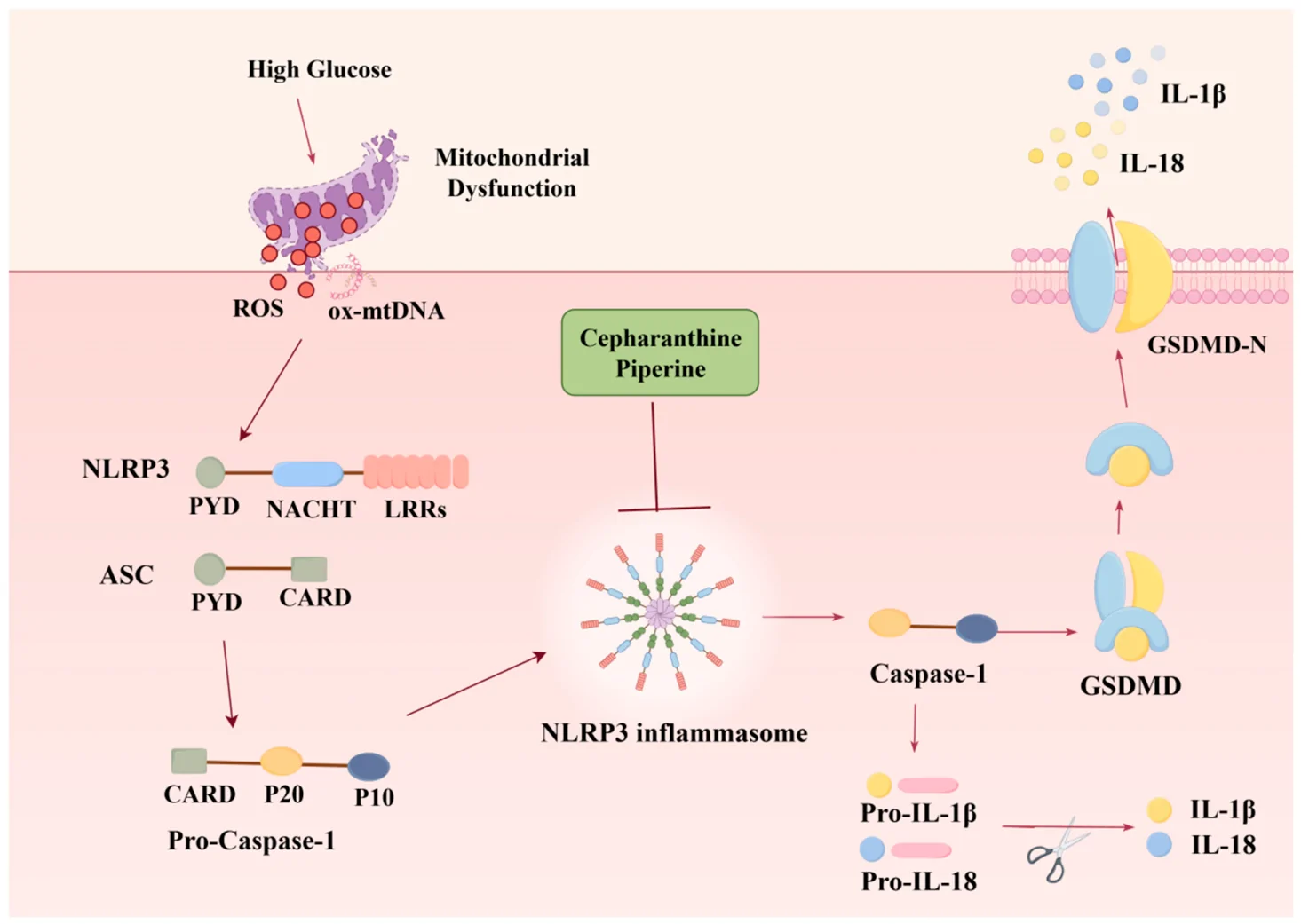
Alkaloids inhibit the activation of the NLRP3 inflammasome signaling pathway. Arrows (→) indicate activation or signal flow; blunt-ended lines (⊣) indicate inhibition; and scissor symbols (✂) indicate enzymatic cleavage. Chemical structures of the compounds involved in this study, such as (C) cepharanthine and (D) piperine, are depicted in Figure 1. Created with Figdraw (by https://www.figdraw.com).

The NLRP3 inflammasome is a pivotal driver of the inflammatory response. Under normal physiological conditions, the NACHT domain of NLRP3 binds to the leucine-rich repeats (LRRs), maintaining the inflammasome in a self-inhibited state [54]. In a high-glucose environment induced by DN, mitochondrial dysfunction leads to the release of ROS and oxidized mitochondrial DNA (ox-mtDNA) into the cytoplasm. These molecules act as danger-associated molecular patterns (DAMPs) and are directly recognized by the NLRP3 sensor in the cytoplasm, inducing a conformational change in the NLRP3 protein. This change lifts the self-inhibitory state, exposing the NACHT domain and triggering oligomerization [55,56,57]. The oligomerization causes the PYD domain at the N-terminus of NLRP3 to aggregate and form a scaffold, which subsequently recruits the ASC adaptor protein containing the PYD (N-terminus) and CARD (C-terminus) domains. The CARD domain of ASC then recruits and activates the pro-caspase-1 precursor (pro-Caspase-1), which consists of an N-terminal CARD, a central p20, and a C-terminal p10, thereby completing inflammasome assembly. Activated caspase-1 cleaves the pro-interleukin-1β (pro-IL-1β) and pro-interleukin-18 (pro-IL-18) precursors, promoting the maturation of IL-1β and IL-18. Simultaneously, caspase-1 cleaves gasdermin D (GSDMD), releasing its N-terminal domain, which forms pores in the lipid membrane, allowing the mature forms of IL-1β and IL-18 to be secreted into the extracellular space and to mediate downstream inflammatory responses [58,59,60] (see Figure 3). The activation of the NLRP3 inflammasome links misfolded proteins, mitochondrial stress, chronic inflammation, and apoptosis in DN, rendering it a highly promising therapeutic target [61,62].

Cepharanthine, a bisbenzylisoquinoline alkaloid derived from the roots of *Stephania japonica* (Thunb.) Miers., which belongs to the Stephania genus in the Menispermaceae family, exhibits notable anti-inflammatory and antioxidant pharmacological properties [63]. It directly interacts with the NLRP3 protein, revealing its ubiquitination site and recruiting the E3 ubiquitin ligase ARIH2. This interaction facilitates K48-linked polyubiquitination of NLRP3, a process specifically recognized by the 26S proteasome, triggering the degradation of NLRP3 through the proteasomal pathway and thereby negatively regulating the assembly and function of the NLRP3 inflammasome [64]. Piperine, a piperidine alkaloid extracted from the mature fruits of black pepper *Piper nigrum*, which belongs to the genus Piper in the family Piperaceae, exhibits various pharmacological activities, including antioxidant and anti-inflammatory effects [65]. Its molecular structure features an amide bond and a benzodioxol ring, the latter enhancing its interaction with NLRP3 by forming hydrogen bonds at the lysine 232 (Lys232) site, consequently inhibiting NLRP3 inflammasome assembly [66]. This study investigated a DN model induced in SD rats through a single intraperitoneal injection of STZ at a dosage of 50 mg/kg. The findings demonstrated that the combination of piperine (30 mg/kg) and cepharanthine (10 mg/kg), administered via intraperitoneal injection once daily for eight consecutive weeks, significantly reduced blood glucose levels, Scr, BUN, the kidney-to-body weight ratio, and urinary protein excretion in DN rats. Additionally, it alleviated pathological changes, including thickening of the glomerular basement membrane and tubular vacuolar degeneration, while significantly downregulating the mRNA and protein expression levels of TXNIP and NLRP3 in renal tissue. Furthermore, this treatment reduced the levels of TNF-α and IL-1β in renal tissue. TXNIP plays a critical role in activating the NLRP3 inflammasome, and its downregulation can inhibit the assembly and activation of the NLRP3 inflammasome, thereby reducing the maturation and secretion of IL-1β. Consequently, this alleviates inflammatory damage to the kidneys in DN [31].

### 4.2. Antioxidant Stress

Oxidative stress serves as a significant trigger for DN and plays a crucial role in its pathological progression. In DN, the excessive production of ROS resulting from oxidative stress can damage various biomolecules, including lipids, proteins, and DNA. This oxidative damage leads to widespread physiological dysfunction, mitochondrial impairment, and inflammatory responses. Concurrently, these inflammatory responses further enhance ROS production, creating a self-perpetuating vicious cycle [67,68]. Ultimately, this process exacerbates glomerulosclerosis, tubulointerstitial fibrosis, and the progressive decline of renal function, propelling DN towards end-stage renal disease.

**Figure 4:**
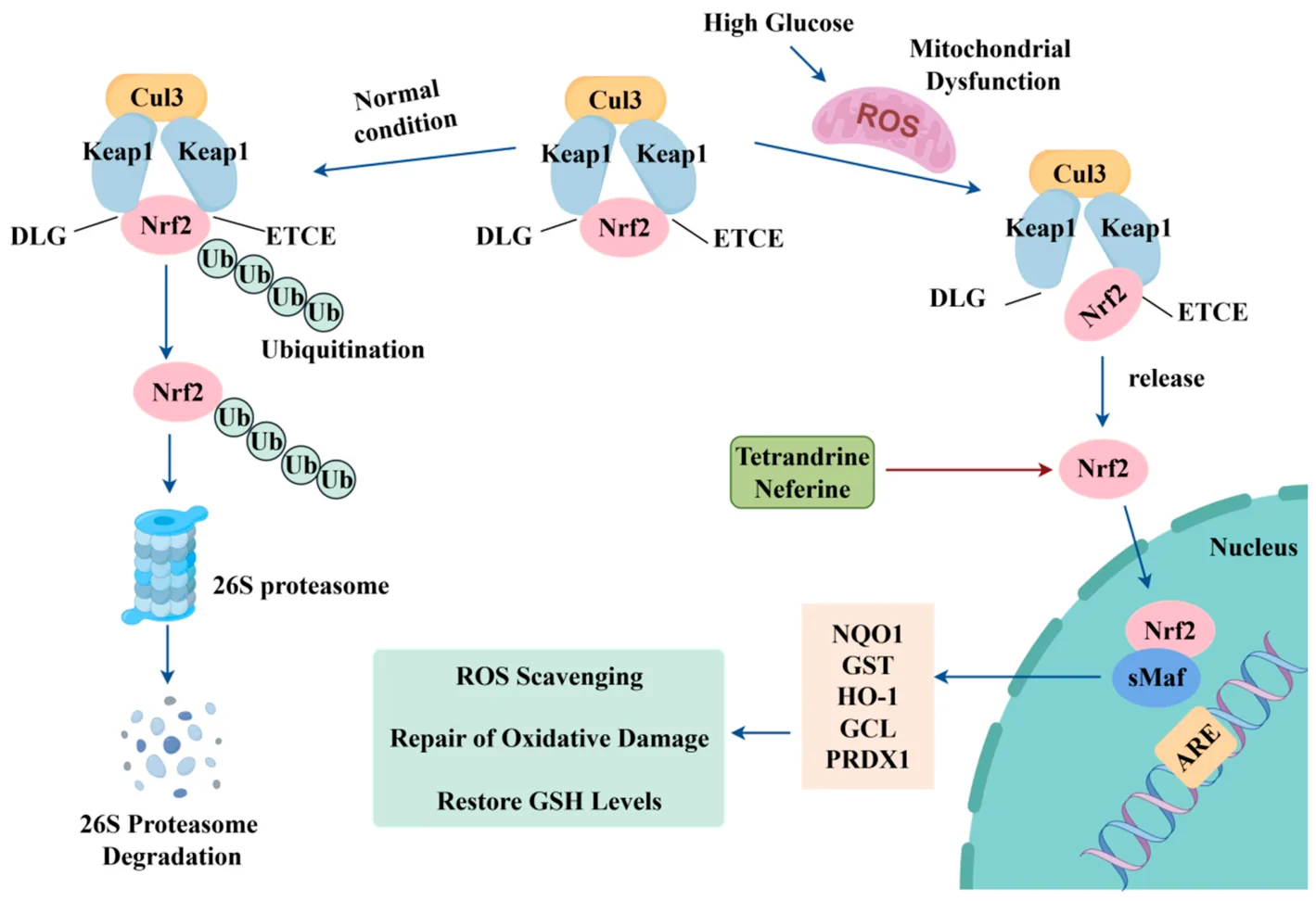
Mechanisms of antioxidant stress response in alkaloid compounds. Arrows (→) indicate activation or signal flow. Chemical structures of the compounds involved in this study, such as (E) tetrandrine and (F) neferine, are depicted in Figure 1. Created with Figdraw (by https://www.figdraw.com).

Nuclear factor erythroid 2-related factor 2 (Nrf2) serves as a pivotal transcription factor that regulates the endogenous cellular antioxidant defense system. Under physiological conditions, Kelch-like ECH-associated protein 1 (Keap1) forms a homodimer that binds to the ETGE and DLG motifs of Nrf2. The Keap1-Nrf2 complex subsequently interacts with the E3 ligase Cullin 3 (Cul3) via the Nrf2 Neh2 domain, resulting in the formation of the Keap1-Cul3-Rbx1 complex. This complex facilitates the ubiquitination of Nrf2, culminating in its degradation by the 26S proteasome [69,70]. In the pathological context of DN, persistent hyperglycemia induces excessive production of ROS, subjecting renal juxtaglomerular cells to a chronic oxidative stress microenvironment. The exposure to oxidative stress modifies key cysteine residues of Keap1 (such as Cys151, Cys273, and Cys288) through ROS or electrophiles, leading to a conformational change in Keap1. Consequently, Nrf2 dissociates from Keap1, thereby evading degradation. The newly synthesized Nrf2 is stabilized and translocates into the cell nucleus, where it forms a heterodimer with the small Maf protein and binds to the Antioxidant Response Element (ARE) in the promoter region of target genes. This interaction initiates the transcriptional expression of Phase II detoxification and antioxidant enzymes, including NAD(P)H:quinone oxidoreductase 1 (NQO1), glutathione S-transferase (GST), heme oxygenase-1 (HO-1), glutamate-cysteine ligase (GCL), and peroxiredoxin 1 (PRDX1), among others [71]. Collectively, these enzymes scavenge ROS, repair oxidative damage, and restore glutathione (GSH) levels, thereby re-establishing cellular redox homeostasis [72] (see Figure 4).

Tetrandrine, a bisbenzylisoquinoline alkaloid, is derived from the dried roots of *Stephania tetrandra* S. Moore., a species within the Stephania genus of the Menispermaceae family. This compound exhibits notable anti-inflammatory and anti-fibrotic pharmacological effects [73]. It consists of two benzylisoquinoline units linked by an ether bond to form a dibenzylisoquinoline dimer. The molecule contains four methoxy groups, and each of the two isoquinoline units can interact with two different binding sites on the Kelch domain of Keap1. This enhances the efficiency of Nrf2 release. A study conducted on a SD rat model of DN, induced by a high-fat diet combined with a single intraperitoneal injection of STZ at a dosage of 35 mg/kg, revealed that intragastric administration of tetrandrine at doses of 5, 15, and 30 mg/kg once daily for eight consecutive weeks resulted in significant reductions in blood glucose, Scr, and proteinuria levels, while also improving renal histopathological damage. The findings indicated that tetrandrine decreased blood glucose, Scr, proteinuria, and BUN levels in DN rats in a dose-dependent manner. Furthermore, it ameliorated renal histopathological damage and significantly reduced levels of malondialdehyde (MDA) and lactate dehydrogenase (LDH). Concurrently, tetrandrine increased superoxide dismutase (SOD) activity and inhibited the production of ROS. Additionally, it activated the Nrf2/HO-1 signaling pathway, resulting in the upregulation of the p-Nrf2/Nrf2 ratio and HO-1 expression. This mechanism alleviated oxidative stress damage and exerted a nephroprotective effect [32].

Neferine is a dibenzylisoquinoline alkaloid extracted from the green embryo, commonly referred to as the ‘lotus seed heart’, of the mature seeds of the lotus *Nelumbo nucifera*, which belongs to the Nelumbo genus within the Nymphaeaceae family. This compound exhibits various pharmacological effects, including anti-inflammatory, anti-fibrotic, and antioxidant properties [74]. Its molecular structure is characterized by two benzylisoquinoline units linked by an ether bond, which contain phenolic hydroxyl and methoxy groups. The dibenzylisoquinoline skeleton underpins the molecule’s antioxidant activity and its capacity to bind to the Kelch domain of Keap1, thereby facilitating Nrf2 activation. The phenolic hydroxyl group plays a crucial role in scavenging free radicals and can interact with cysteine residues in Keap1 through hydrogen bonding, thus promoting the release of Nrf2. A study utilizing a C57BL/6J mouse model of DN, induced by a high-fat diet combined with a single intraperitoneal injection of STZ (100 mg/kg), demonstrated that the intragastric administration of neferine at doses of 60, 120, and 240 mg/kg once daily for 12 consecutive weeks significantly reduced blood glucose levels, kidney weight index, Scr, BUN, and urinary albumin levels in DN mice. Additionally, neferine alleviated renal histopathological damage and reduced MDA levels in renal tissue in a dose-dependent manner. Furthermore, it enhanced the activities of SOD and glutathione peroxidase (GSH-Px) and upregulated the expression of Nrf2 and HO-1 proteins in renal tissue. In vitro studies indicated that human mesangial cells (HMCs), when co-treated with 1600.5 μM (≈1 mg/mL, MW 624.8) neferine and 25 mM high glucose for 48 h, exhibited increased expression levels of Nrf2 and HO-1 proteins [33]. These findings suggest that neferine exerts protective effects by alleviating oxidative stress-induced kidney damage through the activation of the Nrf2/HO-1 signaling pathway.

### 4.3. Anti-Renal Fibrosis

**Figure 5:**
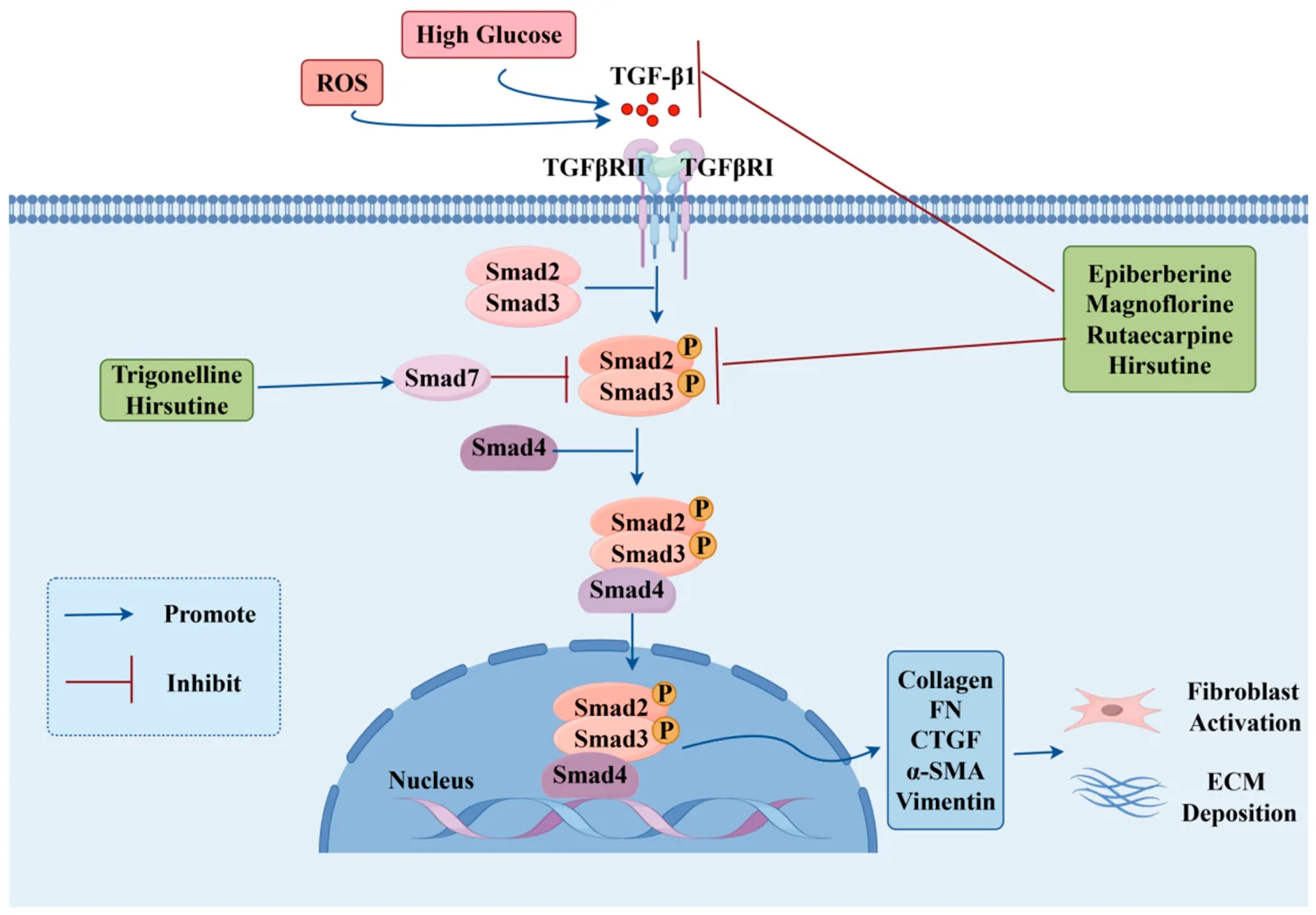
Mechanisms of action of alkaloids in inhibiting renal fibrosis. Arrows (→) indicate activation or signal flow, while blunt-ended lines (⊣) indicate inhibition. Chemical structures of the compounds involved in this study, such as (G) epiberberine, (H) magnoflorine, (I) trigonelline, (J) rutaecarpine, and (K) hirsutine, are depicted in Figure 1. Created with Figdraw (by https://www.figdraw.com).

Renal fibrosis is a pathological process characterized by the excessive production and accumulation of ECM in the kidneys. Specific manifestations include glomerulosclerosis and tubulointerstitial fibrosis [75,76]. The transforming growth factor-β (TGF-β) family comprises three subtypes, TGF-β1, TGF-β2, and TGF-β3, with TGF-β1 being the primary pathogenic subtype in renal fibrosis and considered the core factor driving this process [77,78]. TGF-β1-mediated pro-fibrotic signaling is primarily transmitted through the Smad-dependent classical pathway, which is subject to bidirectional regulation. Receptor-regulated Smad2 and Smad3 transmit pro-fibrotic signals downstream, while inhibitory Smad7 can directly bind to the activated type I TGF-β receptor (TGFβRI), competitively blocking the phosphorylation of Smad2/3 and subsequent Smad complex assembly, thus terminating signal transduction [79]. Under pathological conditions such as DN, high glucose, and ROS directly activate TGF-β1, promoting its binding to the type II TGF-β receptor (TGFβRII), which subsequently activates TGFβRI. This leads to a significant downregulation of Smad7 expression, rendering the negative feedback mechanism ineffective. Under the influence of these dual effects, Smad2 and Smad3 undergo sustained phosphorylation [80]. Phosphorylated Smad2 and Smad3 then bind to Smad4 to form a Smad complex, which translocates to the cell nucleus to regulate the transcription of target genes such as collagen, fibronectin (FN), connective tissue growth factor (CTGF), α-smooth muscle actin (α-SMA), and Vimentin. This ultimately promotes excessive ECM synthesis and deposition, fibroblast activation, and the epithelial–mesenchymal transition (EMT) process, thereby driving the onset and progression of renal fibrosis [81] (see Figure 5).

Epiberberine is a quaternary ammonium isoquinoline alkaloid derived from the dried rhizomes of *Coptis chinensis* Franch. It is an isomer of berberine, possessing identical molecular formulas to its parent compound; the only distinction lies in the interchange of the positions of the methylenedioxy ring and the two methoxy groups on the aromatic skeleton. In berberine, the C-2 and C-3 positions of ring A are occupied by a methylenedioxy ring, whereas the C-9 and C-10 positions of ring D are occupied by dimethoxy groups. In epiberberine, this arrangement is reversed. Although this alteration in substituent position does not disrupt the planar conjugated molecular framework, it reshapes the spatial distribution of hydrogen-bonding acceptors and hydrophobic regions on the molecular surface. This modification leads to an increase in dipole moment and enhanced lipophilicity. Consequently, the oral bioavailability of epiberberine (approximately 14%) is significantly higher than that of berberine (less than 1%). A study found that when 7-week-old male db/db mice, which exhibit spontaneous diabetes, were administered epiberberine at doses of 50, 100, and 200 mg/kg via intragastric administration once daily for eight consecutive weeks, the results indicated that epiberberine reduced fasting blood glucose, TC, TG, and LDL-C levels in a dose-dependent manner. Additionally, it increased HDL-C levels and significantly lowered Scr, BUN, 24 h urinary albumin, and UACR levels. Furthermore, it alleviated pathological changes, including thickening of the glomerular basement membrane, expansion of the mesangial matrix, and collagen deposition. These results indicate that epiberberine exerts a pronounced nephroprotective effect while regulating blood glucose and lipid levels. Concurrently, it downregulated the protein expression levels of TGF-β1, Smad2, and Col-IV in renal tissue in a dose-dependent manner, thereby inhibiting the activation of the TGF-β/Smad2 signaling axis. In in vitro experiments, mouse glomerular mesangial cells (SV40 MES 13) were exposed to 30 mM glucose for 24 h and simultaneously treated with epiberberine at doses of 20, 40, and 80 μM. The results demonstrated that epiberberine significantly reduced TGF-β1 and Smad2 protein expression in glucose-induced SV40 MES 13 cells [34].

Magnoflorine is a benzylisoquinoline alkaloid derived from the dried flower buds of *Magnolia biondii* Pamp., a species within the Magnolia genus of the Magnoliaceae family. It has demonstrated antioxidant, antihypertensive, anti-inflammatory, and antidiabetic properties [82,83]. The compound features a tetrahydroapomorphine skeleton characterized by a quaternary ammonium nitrogen and two phenolic hydroxyl groups. The rigidity and planarity of this apomorphine-type tetrahydro skeleton facilitate binding to target proteins such as KDM3A and TGF-β receptors, thereby alleviating fibrosis and serving as the structural basis for its anti-fibrotic activity. In an SD rat model of DN induced by a single intraperitoneal injection of STZ (70 mg/kg), the study found that intragastric administration of magnoflorine at a dose of 100 mg/kg once daily for 30 consecutive days significantly reduced serum levels of BUN, Scr, and Cys-C, while alleviating renal histopathological damage in DN rats. This damage included vacuolar degeneration of renal tubular epithelial cells, infiltration of inflammatory cells, and collagen deposition in the renal interstitium. Furthermore, magnoflorine significantly downregulated TGF-β1 expression and Smad2/3 phosphorylation levels in renal tissue by inhibiting the TGF-β1/Smad2/3 signaling pathway. In vitro studies indicated that treatment with 25 mM high glucose and 292 μM (≈100 μg/mL, MW 342) magnoflorine for 48 h resulted in significantly reduced expression levels of FN, α-SMA, Col-IV, and TGF-β1 in SV40 MES 13 cells. Additionally, knocking down TGF-β1 expression using siRNA abolished the inhibitory effect of magnoflorine on the TGF-β1/Smad2/3 pathway and its regulation of cell viability [35].

Trigonelline is a pyridine alkaloid extracted from the dried, mature seeds of *Trigonella foenum-graecum* L., a member of the Trigonella genus in the legume family. This compound exhibits various pharmacological effects, including hypoglycemic and hypolipidemic actions [84]. Its structure consists of a pyridinium ring linked to a carboxymethyl group (-CH_2_-COOH), making it a simple N-methylated derivative of nicotinic acid. The carboxymethyl group serves as the key functional group responsible for its anti-fibrotic activity. It forms hydrogen bonds with the MH2 domain of Smad7, thereby enhancing the binding affinity between the molecule and the target protein. This interaction stabilizes the active conformation of Smad7 and extends its half-life. As a result, the transcriptional upregulation of Smad7 is promoted, leading to an anti-fibrotic effect. A study utilizing the spontaneous diabetic db/db mouse model demonstrated that intragastric administration of trigonelline at doses of 50 and 100 mg/kg once daily for eight consecutive weeks significantly reduced Scr, BUN, and UACR levels. Additionally, trigonelline alleviated renal interstitial fibrosis and thickening of the renal tubular basement membrane while downregulating the mRNA and protein expression of the renal injury marker Kim-1. In an in vitro study, the human proximal renal tubular epithelial cell line (HK-2) was treated simultaneously with 80 μM trigonelline and 50 mM high glucose for 36 h. The results indicated that trigonelline significantly reversed the upregulation of vimentin and the downregulation of E-cadherin induced by high glucose, thereby inhibiting the EMT process. The results indicated that trigonelline significantly reversed the upregulation of vimentin and downregulation of E-cadherin induced by high glucose, thereby inhibiting the EMT process. Furthermore, trigonelline was found to upregulate the mRNA and protein expression of Smad7 in renal tissue and HK-2 cells in a dose-dependent manner, significantly reducing the levels of TGFβRI, p-Smad2, and p-Smad3. However, it did not significantly affect total TGF-β1 levels. This indicates that trigonelline may inhibit the TGF-β/Smad pathway by upregulating Smad7 rather than by directly reducing TGF-β1 levels. Moreover, knocking down Smad7 expression using siRNA significantly reversed the regulatory effects of trigonelline on TGFβRI, p-Smad3, and EMT markers (E-cadherin and Vimentin), confirming that the anti-EMT and anti-fibrotic effects of trigonelline are dependent on Smad7 [36]. In addition to directly regulating the TGF-β/Smad pathway, trigonelline can inhibit fibrosis via the Wnt/β-catenin pathway, indicating that it targets multiple mechanisms. In a separate in vitro experiment, human glomerular mesangial cells (HMCs) were treated with 50, 100, or 200 μM of trigonelline alongside 30 mM high glucose for 48 h. The results demonstrated that trigonelline inhibited the excessive proliferation of mesangial cells induced by high glucose in a dose-dependent manner, significantly reducing the secretion and expression of FN and Col-IV. Furthermore, the fenugreek alkaloid was found to downregulate the levels of Wnt4, Wnt5a, and the nuclear proteins β-catenin and TCF4, while concurrently decreasing the expression of the downstream cyclins Cyclin D1 and CDK4. This suggests that trigonelline inhibits ECM gene transcription by blocking the activation of the Wnt/β-catenin pathway. Additionally, the use of a β-catenin overexpression plasmid reversed the inhibitory effects of trigonelline on FN and Col-IV secretion and expression, thereby confirming that trigonelline downregulates their expression in mesangial cells by inhibiting Wnt/β-catenin signaling, which reduces ECM accumulation and slows the progression of glomerulosclerosis in DN [37].

Rutaecarpine is a quinazoline alkaloid derived from the dried, mature fruits of *Evodia rutaecarpa* (Juss.) Benth., a plant belonging to the Euodia genus within the Rutaceae family. It exhibits a range of pharmacological effects, including antiplatelet, antioxidant, anti-inflammatory, lipid-lowering, antithrombotic, and vasodilatory actions [85]. Its molecular structure features a rigid, fused five-ring skeleton comprising 14 nitrogen atoms (N-14) and five carbonyl groups (C=O). This highly planar and rigid five-ring structure allows it to stably embed itself into the ATP-binding pocket of the CK2α protein kinase subunit through π-π stacking and hydrophobic interactions, thereby directly inhibiting CK2α kinase activity. Additionally, it disrupts the assembly of the TGF-β receptor-associated signaling complex via hydrophobic interactions. A study utilizing a db/db spontaneous diabetic mouse model demonstrated that intragastric administration of rutaecarpine at doses of 5, 10, and 20 mg/kg once daily for 12 consecutive weeks significantly mitigated renal histopathological damage. This included reductions in glomerular hypertrophy, mesangial matrix expansion, glomerular basement membrane thickness, and mesangial ECM deposition, all in a dose-dependent manner. In vitro experiments were conducted on SV40 MES 13 cells treated with 0.5, 1, and 2 μM of evodiamine, alongside a 30 mM high-glucose solution, for a duration of 24 h. The results indicated that evodiamine exhibited a concentration-dependent inhibition of cell proliferation. Furthermore, rutaecarpine effectively blocked the activation of the TGF-β1/Smad3 signaling pathway, leading to a significant downregulation of FN, Col-IV, α-SMA, and TGF-β1 protein expression in both renal tissue and SV40 MES 13 cells. It also inhibited Smad3 phosphorylation and reduced ECM deposition in glomerular mesangial cells associated with DN [38].

Hirsutine, a monoterpene indole alkaloid, is extracted from the dried, hooked stems of *Uncaria rhynchophylla* (Miq.) Miq. ex Havil., a plant belonging to the Uncaria genus within the Rubiaceae family. It exhibits various pharmacological activities, including anti-inflammatory, antioxidant, antidiabetic, and vasodilatory effects [86]. Its molecular structure features a tetracyclic corynanthe-type skeleton that contains a methyl ester group (-COOCH_3_) and an α,β-unsaturated ester. The multiple ring structures and hydrophobic regions of the tetracyclic corynanthe-type skeleton facilitate binding to the hydrophobic pocket of TGFβRI. The methyl ester group (-COOCH_3_) can form hydrogen bonds with TGFβRI, enhancing binding affinity and competitively inhibiting TGFβRI kinase activity. This inhibition blocks TGF-β1-induced Smad2/3 phosphorylation and nuclear translocation, ultimately exerting a renoprotective effect. The study demonstrated that in a spontaneous diabetic db/db mouse model, the daily intragastric administration of 20 mg/kg of hirsutine for eight consecutive weeks significantly reduced 24 h urinary protein excretion, UACR, BUN, and Scr levels. Furthermore, it markedly alleviated vacuolar degeneration of renal tubular epithelial cells and collagen fiber deposition, while also improving renal histopathological damage. Concurrently, hirsutine significantly downregulated the expression of TGF-β1, p-Smad2, and p-Smad3 proteins in renal tissue by inhibiting the activation of the TGF-β/Smad signaling pathway, thereby alleviating renal fibrosis associated with DN. However, the co-administration of SRI-011381, a TGF-β signaling pathway agonist, significantly reversed the renoprotective effects of hirsutine and its inhibitory effects on the TGF-β/Smad signaling pathway [39].

### 4.4. Regulating Autophagy

Autophagy is an evolutionarily conserved, lysosome-dependent degradation pathway that plays a crucial role in the specific clearance of damaged organelles, such as mitochondria and the endoplasmic reticulum, as well as abnormally aggregated proteins, including AGE-modified proteins and misfolded proteins, within cells. This process is essential for maintaining intracellular homeostasis [87,88]. In the early stages of DN, the autophagy pathway can be compensatorily activated by cells, exerting a clear endogenous protective effect on glomerular podocytes and renal tubular epithelial cells. However, prolonged exposure to a high-glucose pathological microenvironment leads to impaired autophagic flux. This impairment is characterized by specific defects in membrane fusion between autophagosomes and lysosomes, as well as deficiencies in lysosomal hydrolytic function. Consequently, there is a substantial intracellular accumulation of autophagy marker substrates, such as p62/SQSTM1, which exacerbates cellular oxidative damage and contributes to the fibrotic phenotype [89].

AMP-activated protein kinase (AMPK) serves as a crucial sensor that regulates energy metabolism and homeostasis in eukaryotic cells, functioning as a significant positive regulator of the autophagy pathway [90]. In the context of DN, prolonged exposure to elevated glucose levels induces mitochondrial dysfunction and diminishes the efficiency of intracellular adenosine triphosphate (ATP) synthesis. This results in a substantial increase in the cytoplasmic adenosine monophosphate (AMP)/ATP ratio. Consequently, this alteration recruits the upstream liver kinase β1 (LKβ1) and the calcium/calmodulin-dependent protein kinase kinase β (CaMKKβ). These two kinases catalyze the phosphorylation of Thr172 on the AMPKα catalytic subunit, thereby fully activating AMPK [91]. Upon activation, AMPK directly targets and phosphorylates the Ser792 site of Raptor, a pivotal subunit of the mammalian target of rapamycin complex 1 (mTORC1). This phosphorylation disrupts the spatial conformation of the mTORC1 complex, indirectly inhibiting mTORC1 kinase activity [92]. When mTORC1 activity is inhibited, the downstream autophagy initiation signal, Unc-51-like autophagy-activating kinase 1 (ULK1), becomes dephosphorylated and activated. This activation leads to the activation of autophagy-related protein 14 (ATG14), Beclin-1, and VPS34, thereby initiating the nucleation and elongation of the autophagosome membrane. Microtubule-associated protein 1 light chain 3 (LC3) is subsequently phosphorylated by a cascade of ubiquitin-like modifying enzymes, including autophagy-related protein 4 (ATG4), autophagy-related protein 7 (ATG7), and autophagy-related protein 3 (ATG3), among others. This process anchors LC3 stably to the bilayer membrane structure of the nascent autophagosome [93]. LC3-II on the autophagosome membrane interacts specifically with lysosomal-associated membrane protein 2 (LAMP2) on the lysosomal membrane surface, mediating precise autophagosome-lysosome fusion. The autophagic substrates enclosed within the lysosomal lumen are degraded by acidic hydrolases into small molecules such as amino acids and fatty acids, which are then released into the cytoplasm via lysosomal membrane transporters, allowing them to re-enter the metabolic cycle [94].

**Figure 6:**
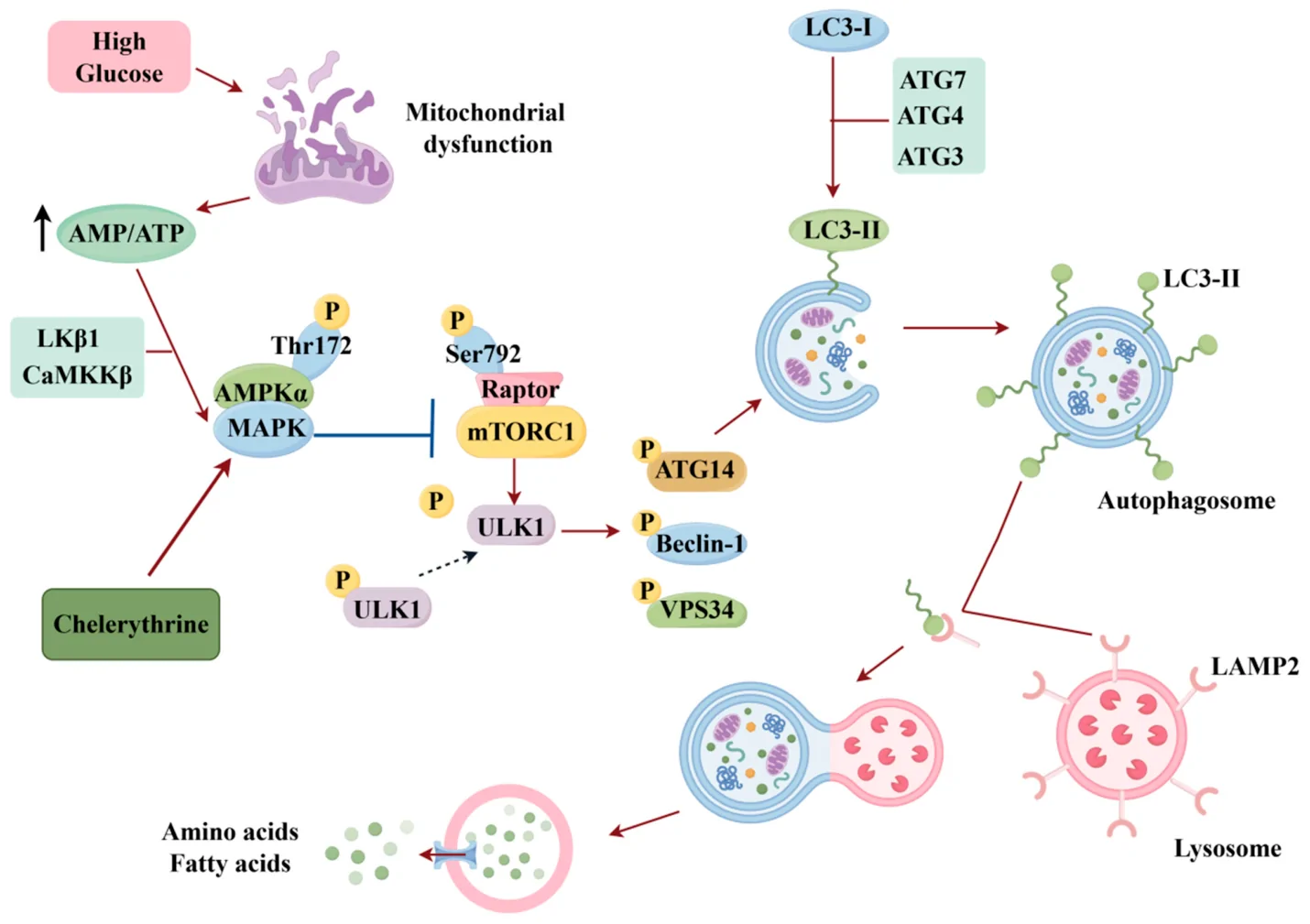
Alkaloids regulate autophagy pathways. Arrows (→) indicate activation or signal flow, while blunt-ended lines (⊣) indicate inhibition. Chemical structures of the compounds involved in this study, such as (L) chelerythrine, are depicted in Figure 1. Created with Figdraw (by https://www.figdraw.com).

In the pathological progression of DN, chronic hyperglycemia significantly inhibits AMPK activity and induces excessive activation of the mTORC1, leading to a global disruption of the autophagy pathway. It also causes downregulation of LAMP2 expression, impaired lysosomal acidification, and suppressed cathepsin activity. These changes result in dysfunction of the LC3-II-LAMP2 axis and disruption of autophagy flux at the fusion-degradation stage [95]. Collectively, these autophagy impairments lead to the accumulation of p62/SQSTM1 and damaged mitochondria, triggering podocyte detachment and apoptosis, as well as renal tubular epithelial cell hypertrophy and interstitial fibrosis. These processes constitute a key mechanism in the progression of DN. Restoring normal intracellular autophagy through targeted strategies can effectively promote mitochondrial autophagy and alleviate endoplasmic reticulum stress mediated by the unfolded protein response. This intervention blocks the aforementioned cascade of events at the cellular level, thereby delaying the pathological progression of DN [96] (see Figure 6).

Chelerythrine, a benzylisoquinoline alkaloid, is derived from the entire plant of greater celandine, *Chelidonium majus* L., which belongs to the Chelidonium genus within the poppy family (Papaveraceae). It exhibits various pharmacological effects, including anti-inflammatory and antibacterial properties [97]. Chelerythrine features a planar benzo[b]pyridine ring system, which includes a methylenedioxy group (C7–C8) and two methoxy groups (C9 and C10), as well as a quaternary ammonium nitrogen. This specific ring structure facilitates chelerythrine’s insertion into the ATP-binding pocket of AMPK, thereby activating the AMPK/mTOR pathway and promoting the restoration of autophagy. The study investigated an SD rat model of DN, induced by a single intraperitoneal injection of STZ at a dose of 55 mg/kg. The findings revealed that intragastric administration of chelerythrine at doses of 10 and 20 mg/kg once daily for 42 consecutive days led to a dose-dependent reduction in fasting blood glucose, kidney index, Scr, BUN, urinary protein, Cys-C, neutrophil gelatinase-associated lipocalin (NGAL), and KIM-1 levels in DN rats. Furthermore, chelerythrine alleviated pathological changes, including glomerular atrophy, basement membrane thickening, and collagen deposition. Mechanistically, chelerythrine activated AMPK and inhibited mTORC1 signaling, significantly increasing the levels of p-AMPK (Thr172) and p-ACC (Ser79) in renal tissue. Additionally, it downregulated the expression of p-mTOR (Ser2448) and p-p70S6K (Thr389), while significantly enhancing the mRNA and protein expression of LC3-II, Beclin-1, and autophagy-related protein 5 (ATG5). This resulted in a reduction in p62 levels and restored autophagic flux, effectively clearing damaged mitochondria [40].

### 4.5. Regulation of Cell Apoptosis

**Figure 7:**
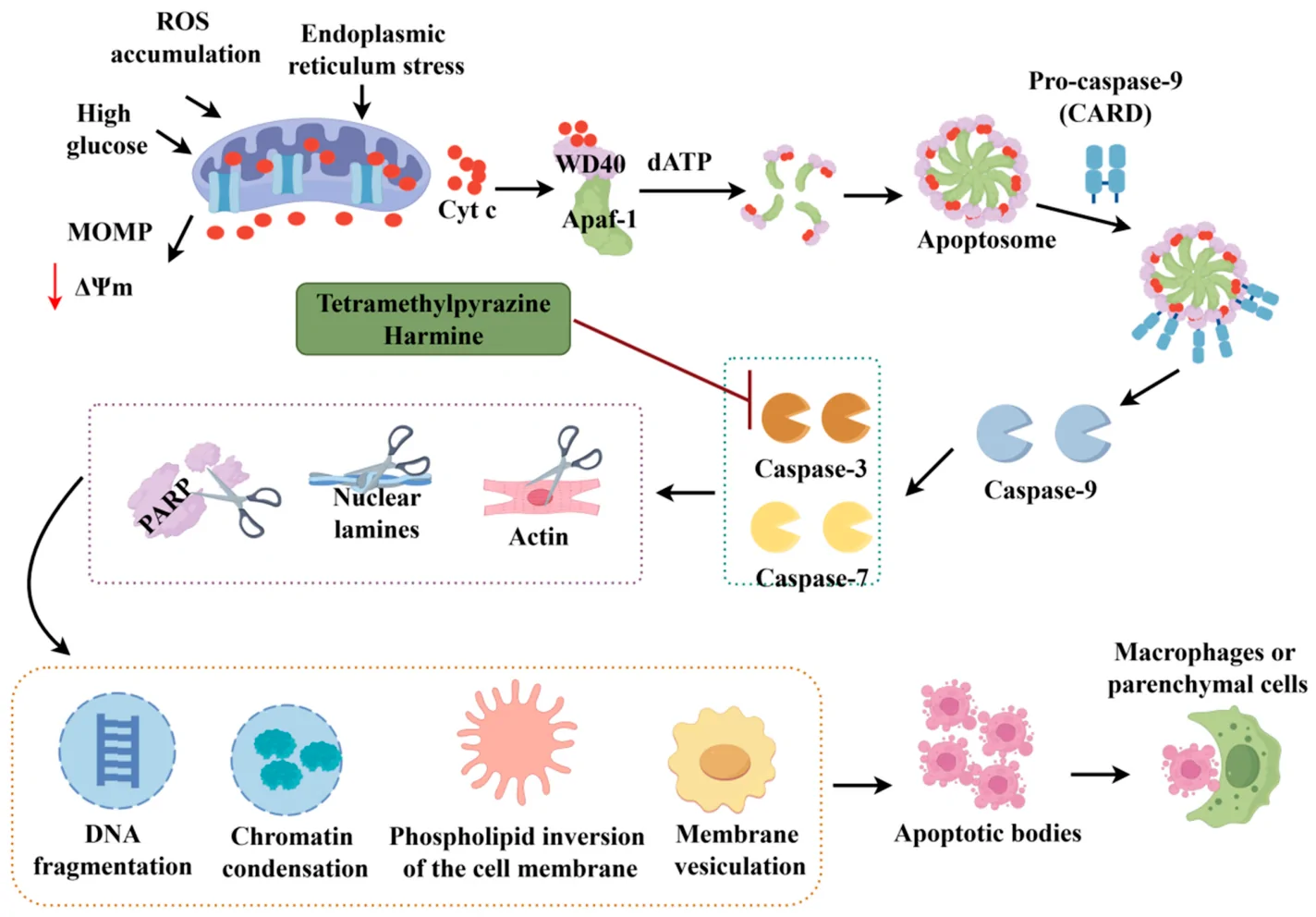
Mechanisms by which alkaloids regulate apoptosis. Arrows (→) indicate activation or signal flow, while blunt-ended lines (⊣) indicate inhibition. Chemical structures of the compounds involved in this study, such as (M) tetramethylpyrazine and (N) harmine, are depicted in Figure 1. Created with Figdraw (by https://www.figdraw.com).

Apoptosis, the primary form of programmed cell death, serves as a critical pathogenic mechanism in the progression of DN. The abnormal and excessive activation of the apoptotic pathway results in the progressive loss of glomerular podocytes and atrophy of renal tubular epithelial cells [98]. Numerous apoptotic stimuli, including high glucose, ROS accumulation, and endoplasmic reticulum stress, have been shown to synergistically induce mitochondrial outer membrane permeabilization (MOMP). This event represents a pivotal rate-limiting phase in the execution of apoptosis, leading to a decline in mitochondrial membrane potential (ΔΨm). Consequently, this decline facilitates the release of a significant number of pro-apoptotic proteins, which are initially localized in the mitochondrial inner membrane space, into the cytoplasm. This process subsequently initiates a multilevel, cascade-amplified, endogenous apoptotic execution program [99]. Among these, cytochrome c (Cyt c) plays a pivotal role as the central initiator of the endogenous apoptotic cascade. It has been demonstrated that Cyt c directly binds to the WD40 repeat sequence of apoptotic protease-activating factor-1 (Apaf-1) through its C-terminal domain. This binding process is further facilitated by the presence of deoxyadenosine triphosphate (dATP), which induces a conformational change in Apaf-1. Consequently, Apaf-1 self-assembles into a functional heptameric complex known as the apoptosome. Through homology-based interactions mediated by its N-terminal CARD domain, this complex selectively recruits the pro-caspase-9 precursor molecule from the cytoplasm, inducing its self-cleavage and activation to yield active caspase-9 with full catalytic functionality. Following this initial activation, activated caspase-9 meticulously activates the downstream effector caspases, namely caspase-3 and caspase-7, through a sequence of enzymatic cleavage reactions [100]. Fully activated caspase-3 and caspase-7 exhibit significant redundancy and collaboratively cleave numerous key intracellular substrate proteins, including poly(ADP-ribose) polymerase (PARP), nucleofil, and actin, along with other cytoskeletal components. This synergistic cleavage event ultimately induces the characteristic morphological changes associated with apoptosis, such as DNA fragmentation, chromatin condensation, phospholipid inversion of the cell membrane, and membrane vesiculation. The resulting apoptotic bodies can be efficiently cleared by neighboring macrophages or parenchymal cells through endocytosis, thereby preventing the inflammatory cascade triggered by the leakage of intracellular contents [68,101] (see Figure 7).

Tetramethylpyrazine, a simple pyrazine alkaloid, is derived from the rhizome of *Ligusticum sinense Chuanxiong*, a plant belonging to the Apiaceae family and the genus Ligusticum. It exhibits antioxidant, anti-inflammatory, and anti-apoptotic properties, while also improving microcirculation [102]. The chemical structure of tetramethylpyrazine features a pyrazine ring with two methyl substituents, where the two nitrogen atoms establish hydrogen bonds with key residues in the ATP-binding pocket of Akt through their lone pairs of electrons. The electron-donating effect of the two methyl groups enhances the electron density of the pyrazine ring, thereby increasing its binding affinity for Akt and activating the Akt signaling pathway. A study utilizing a Wistar rat model of DN, induced by an intraperitoneal injection of nicotinamide (210 mg/kg), followed 15 min later by an injection of STZ (55 mg/kg), demonstrated that intragastric administration of tetramethylpyrazine at doses of 100, 150, and 200 mg/kg, once daily for eight consecutive weeks, resulted in a dose-dependent increase in the activities of SOD and glutathione peroxidase (GSH-Px) in renal tissue and serum. Furthermore, it significantly reduced MDA levels and, by activating the Akt signaling pathway, markedly upregulated the p-Akt/Akt ratio and the expression of the anti-apoptotic protein Bcl-2 in renal tissue. Additionally, it downregulated the p-GSK-3β/GSK-3β ratio and the expression of pro-apoptotic proteins Bax and cleaved caspase-3, thereby alleviating apoptosis in renal tubular epithelial cells and mitigating renal injury in DN [41].

Harmine, a β-carboline-type indole alkaloid, is derived from the seeds of *Peganum harmala* L., a plant belonging to the genus Peganum in the Euphorbiaceae family. It exhibits various pharmacological activities, including antioxidant, anti-inflammatory, and antidiabetic effects [103]. Its structure features a tricyclic, conjugated, planar core that contains 7-methoxy and 1-methyl groups. This tricyclic conjugated planar core engages in π-π stacking interactions with the ATP-binding pocket of ATM kinase and the p53 domain. Such interactions effectively inhibit ATM autophosphorylation, leading to a decrease in p53 phosphorylation levels and thereby blocking the apoptotic pathway. A study utilizing a Wistar rat model of DN, induced by a single intraperitoneal injection of STZ at a dose of 60 mg/kg, demonstrated that daily intraperitoneal administration of harmine at a dosage of 10 mg/kg for two consecutive weeks significantly reduced fasting blood glucose levels, urinary albumin concentrations, and BUN levels in DN rats. Additionally, harmine treatment resulted in increased urinary creatinine levels and mitigated glomerular atrophy, cystic dilatation, tubular degeneration, necrosis, and inflammatory cell infiltration. Furthermore, it improved renal histopathological damage. Concurrently, harmine inhibited the ATM-signal-mediated apoptotic pathway, leading to a significant reduction in the p-ATM/ATM and p-p53/p53 ratios, an upregulation of Bcl-2 expression, and a downregulation of Bax and Caspase-3 expression. This treatment also decreased the Bax/Bcl-2 ratio and suppressed mitochondrial-mediated apoptosis, thereby alleviating kidney damage induced by DN [42].

### 4.6. Resistance to Ferroptosis

Ferroptosis is a recently identified form of programmed cell death that is distinct from apoptosis, autophagy, and necrosis. It is characterized by the accumulation of excessive lipid peroxides driven by intracellular iron ions and is typically associated with various morphological, biochemical, and genetic changes [104]. The mechanisms underlying ferroptosis can be summarized into three interrelated processes: dysregulation of iron metabolism, lipid peroxidation, and dysfunction of glutathione peroxidase 4 (GPX4).

Firstly, regarding disorders of iron metabolism, ferritin, which consists of a heavy chain (ferritin heavy chain, FTH) and a light chain (ferritin light chain, FTL), serves as the primary form of intracellular iron storage. A downregulation of its expression leads to increased iron release, exacerbating iron overload. Concurrently, the impaired function of the iron efflux protein ferroportin (encoded by the SLC40A1 gene) prevents the effective excretion of intracellular iron, further promoting its accumulation. Intracellular free Fe^2+^ reacts with H_2_O_2_ via the Fenton reaction to produce highly reactive hydroxyl radicals (·OH), thereby initiating a lipid peroxidation chain reaction [105].

Secondly, in the context of lipid peroxidation, acyl-CoA synthetase long-chain family member 4 (ACSL4) and lysophosphatidylcholine acyltransferase 3 (LPCAT3) serve as pivotal enzymes in this biochemical process. ACSL4 catalyzes the conversion of free polyunsaturated fatty acids (PUFAs) into polyunsaturated fatty acid-coenzyme A (PUFA-CoA) thioesters. Following this, LPCAT3 facilitates the esterification of the acyl chain of PUFA-CoA into membrane phospholipids, resulting in the formation of polyunsaturated fatty acid-containing phospholipids (PUFA-PL). This process provides a membrane-localized substrate that is directly involved in lipid peroxidation. Conversely, the absence of ACSL4 inhibits the activation of PUFAs and their incorporation into membrane structures, leading to a marked decrease in membrane PUFA-PL levels and, consequently, a reduction in cellular resistance to ferroptosis. Under these circumstances, the cell membrane becomes enriched with PUFA-PL. The PUFA side chains of these phospholipids are susceptible to radical chain auto-oxidation in the presence of ROS and Fe^2+^, resulting in the production of substantial amounts of lipid peroxides (L-OOH). These peroxides ultimately decompose into cytotoxic end products, such as MDA and 4-hydroxynonenal (4-HNE). These cytotoxic products can cross-link membrane proteins and nucleic acids, thereby disrupting the structural integrity of the membrane and ultimately leading to membrane rupture and cell death [106].

Finally, with respect to GPX4 dysregulation, the cystine/glutamate antiporter (System Xc^−^), which comprises the SLC7A11 and SLC3A2 subunits, facilitates the uptake of extracellular cystine and its subsequent intracellular conversion, thereby providing a crucial source of cystine for GSH synthesis. GSH, a tripeptide synthesized through a two-step process catalyzed by GCL and glutathione synthetase (GS), is derived from cysteine, glutamate, and glycine. It acts as an essential cofactor for the catalytic activity of GPX4, the only selenoprotein in mammals capable of directly reducing membrane-bound lipid peroxides. The selenocysteine residue located in its active site is critical for its catalytic function. GPX4 catalyzes the reduction of L-OOH to non-toxic lipid alcohols (L-OH), effectively terminating lipid peroxidation chain reactions. When the function of System Xc^−^ is suppressed or GSH is depleted, the activity of the GPX4 enzyme significantly declines, resulting in the accumulation of lipid peroxides and ultimately triggering ferroptosis [107].

**Figure 8:**
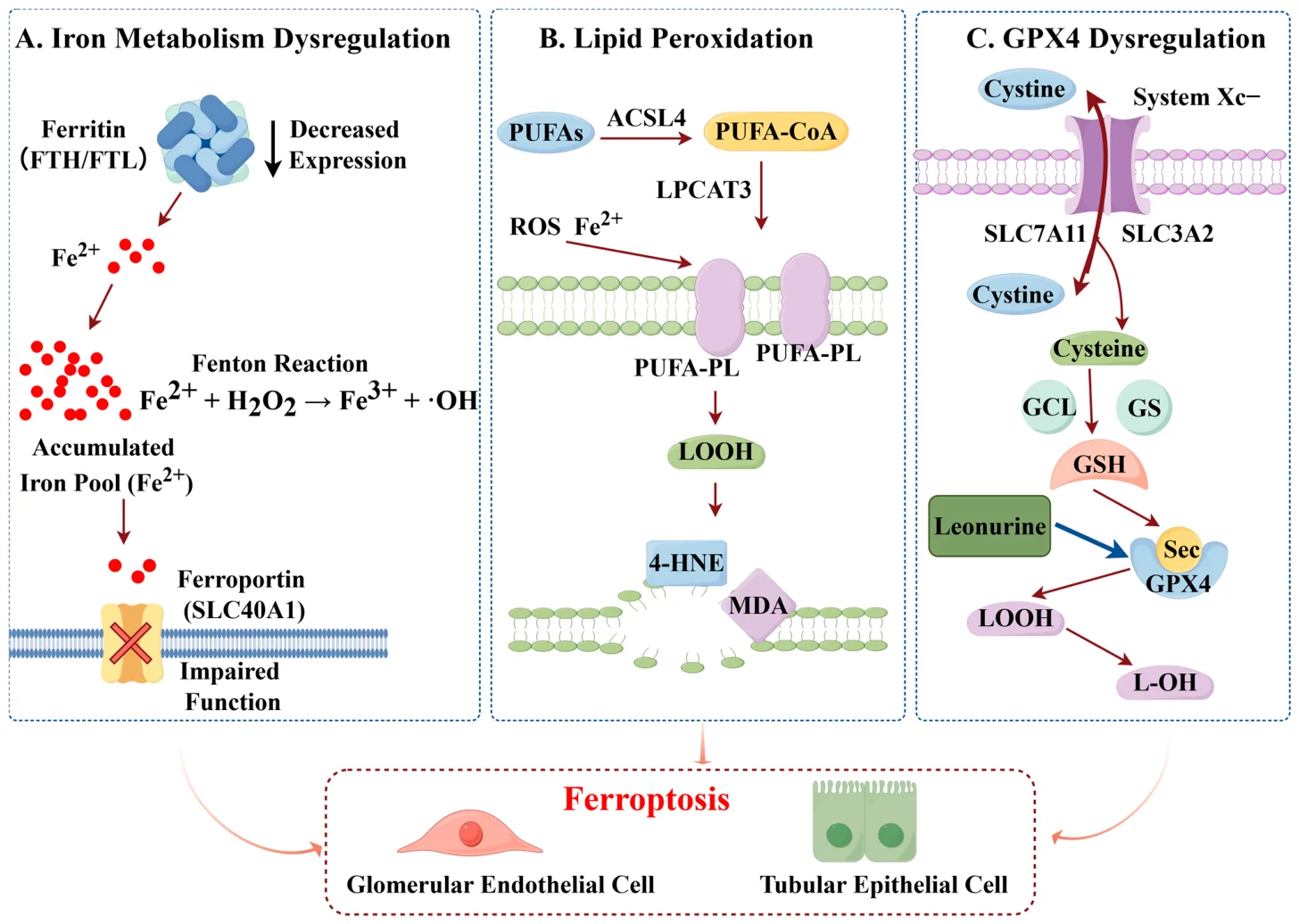
Mechanisms of the anti-ferroptosis effects of alkaloids. Arrows (→) indicate activation or signal flow. Chemical structures of the compounds involved in this study, such as (O) leonurine, are depicted in Figure 1. Created with Figdraw (by https://www.figdraw.com).

In DN, high-glucose-induced disturbances in iron metabolism, lipid peroxidation, and GPX4 dysregulation collectively promote ferroptosis in glomerular endothelial cells and renal tubular epithelial cells, leading to a progressive loss of renal function (see Figure 8). Targeting ferroptosis has emerged as a promising therapeutic strategy for DN.

Leonurine, a phenolic organic amine alkaloid, is extracted from the aerial parts of *Leonurus japonicus* Houtt., a plant belonging to the Lamiaceae family and the genus Leonurus. It exhibits various pharmacological activities, including anti-inflammatory, antioxidant, apoptosis-regulating, and vasoconstriction-inhibiting effects [108]. The guanidinium functional group and the phenolic hydroxyl group in its molecular structure are the core active components. Specifically, the guanidinium group can directly chelate intracellular free Fe^2+^, thereby blocking the initiation of the Fenton reaction and inhibiting the lipid peroxidation chain reaction at its source. Meanwhile, the phenolic hydroxyl group can directly scavenge lipid reactive oxygen species, thereby enhancing the enzymatic activity of GPX4 and maintaining the functional homeostasis of the System Xc^−^-GSH-GPX4 antioxidant axis. A study utilizing a C57BL/6J mouse model of DN, induced by a high-fat diet combined with intraperitoneal injection of STZ (50 mg/kg) over five consecutive days, demonstrated that intragastric administration of leonurine (60 mg/kg) once daily for 12 weeks significantly restored the expression of GPX4, FTL, and FTH proteins in renal tissue. Additionally, it reduced iron content and MDA levels, increased GSH levels, and alleviated glomerular iron deposition. In vitro studies treated human umbilical vein endothelial cells (HUVECs) simultaneously with 40 μM leonurine and 30 mM high glucose for 24 h. The results indicated that leonurine significantly decreased ROS and MDA levels, increased GSH levels, restored mitochondrial membrane potential, and upregulated the expression of endothelial function markers (CD31 and VE-cadherin). Furthermore, it promoted cell migration and significantly reversed the downregulation of GPX4, FTH, and FTL protein expression induced by high glucose. Leonurine also significantly upregulates Nrf2 protein expression by activating the Nrf2/GPX4 axis. As a transcription factor, Nrf2 translocates into the nucleus, binds to the ARE sequence, and initiates GPX4 gene transcription, thereby restoring GPX4 protein levels. Following the knockdown of GPX4 using siRNA, the protective effects of leonurine on iron levels, GSH, MDA, and other ferroptosis markers, as well as on endothelial function markers (CD31 and VE-cadherin) and mitochondrial membrane potential, were significantly reversed. This observation indicates that GPX4 is a critical target through which leonurine exerts its anti-ferroptotic effects. Furthermore, the protective effects of leonurine on the aforementioned markers were similarly significantly reversed following treatment with the Nrf2 inhibitor ML385, thereby confirming that Nrf2 mediates the upregulation of GPX4 induced by leonurine. Collectively, these results suggest that leonurine exerts its anti-ferroptotic effects via the Nrf2/GPX4 axis [43].

## 5. Integration of Cross-Class Structure–Activity Relationships of Alkaloids Against DN

Chapter 4 elucidates the structural characteristics of representative alkaloids and their corresponding mechanisms of action. Building upon this foundation, the current chapter integrates cross-class structure–activity relationships. The renal protective effects of alkaloids against DN are primarily influenced by the parent nucleus, characteristic pharmacologically active groups, types of substituents, and stereochemistry. Quaternary ammonium cations predominantly facilitate electrostatic anchoring and are frequently associated with the inhibition of anti-inflammatory and anti-fibrotic signaling pathways. Free phenolic hydroxyl groups serve as hydrogen-bond donors and scavenge ROS, often correlating with antioxidant and anti-ferroptotic effects. Methylenedioxy, methoxy, and conjugated systems modulate molecular planarity, hydrophobicity, and electron distribution, while fused rings, ether bridges, spiro rings, amides, and ester bonds maintain conformation and influence target pocket compatibility. These patterns indicate that, despite significant structural differences across various classes of alkaloids, each can intervene in key pathological processes of DN, including inflammation, oxidative stress, fibrosis, autophagy, apoptosis, and ferroptosis, through the ‘skeleton–pharmacophore–target pocket’ matching model. Alkaloids from different chemical classes exhibit distinct structure–activity relationship characteristics, which are summarized in Table 3.

Among the various chemical classes, isoquinoline alkaloids demonstrate the most diverse SAR characteristics. Both berberine (Figure 1A) and epiberberine (Figure 1G) possess a protoberberine core, featuring a quaternary ammonium N^+^ group, a methylenedioxy group, and a methoxy group. Variations in their substitution positions modify the distribution of hydrogen bonds and hydrophobic interactions on the molecular surface, consequently influencing their affinities for TLR4/NF-κB and TGFβRI. Magnoflorine (Figure 1H) contains a monomeric benzylisoquinoline core, a positively charged quaternary ammonium group, and polyphenolic hydroxyl groups, which collectively inhibit TGF-β/Smad signaling. Chelerythrine (Figure 1L) activates AMPK-mTOR-mediated autophagy through its planar benzo[b]pyridine-fused ring and quaternary ammonium cation. In the case of dibenzylisoquinoline derivatives, the dimeric core and aromatic ether bridges dictate the conformation, while the quantity of ether bridges, along with the arrangement of phenolic hydroxyl groups and methoxy/methylenedioxy groups, distinguishes their biological activities. The free phenolic hydroxyl group in neferine (Figure 1F) is crucial for its Nrf2-mediated antioxidant activity. Sinomenine (Figure 1B) relies on its intact morpholine skeleton and the C-4 phenolic hydroxyl group to inhibit NF-κB, while the C-6 ketone group and C-7 methoxy group enhance binding affinity.

Among the alkaloids derived from pyridine, piperidine, and pyrazine, the N-methylpyridinium cation in trigonelline (Figure 1I) forms a zwitterion with the C-3 carboxylate group, which upregulates Smad7 and inhibits the TGF-β1/Smad pathway. Piperine (Figure 1D) synergistically inhibits the NLRP3 inflammasome through its piperidinamide group, methylenedioxy aromatic ring, and conjugated alkene chain. Tetramethylpyrazine (Figure 1M) exhibits moderate lipophilicity due to the symmetrical tetramethyl substitution on its pyrazine ring and exerts anti-apoptotic effects by regulating the PI3K/Akt/GSK-3β pathway. Among the quinazoline and indole alkaloids, rutaecarpine (Figure 1J) inhibits the TGF-β1/Smad3 pathway through its planar, conjugated quinazoline–indole fused core and the N-14 methyl group. In the tetracyclic monoterpene indole skeleton of hirsutine (Figure 1K), the indole N-H group and the C-17 methoxycarbonyl group jointly upregulate Smad7, thereby blocking the TGF-β/Smad pathway. In the β-carboline core of harmine (Figure 1N), the free indole N-H group and the C-7 methoxy group activate AMPK, thereby inhibiting the ATM-p53 apoptotic signaling pathway. The organic amine leonurine (Figure 1O) inhibits ferroptosis by stabilizing the GPX4/GSH axis via its guanidinium group and scavenging ROS through the C-4 phenolic hydroxyl group.

Overall, the key structural units that frequently recur include quaternary ammonium nitrogen cations, free phenolic hydroxyl groups, methylenedioxy groups, methoxy groups, and fused-ring or ether bridge frameworks. These units are associated with various activities, such as anti-inflammatory, antioxidant, target-binding, and conformational stabilization. Current SAR inferences are primarily derived from molecular docking studies, derivative analyses, and pharmacological phenotype correlations (refer to Supplementary Note S1 for a comprehensive discussion of SAR). However, there remains a notable deficiency in site-specific structural modifications and standardized IC_50_ data for the DN model. Consequently, the aforementioned patterns necessitate further experimental validation.

## 6. Conclusions and Outlook

**Figure 9:**
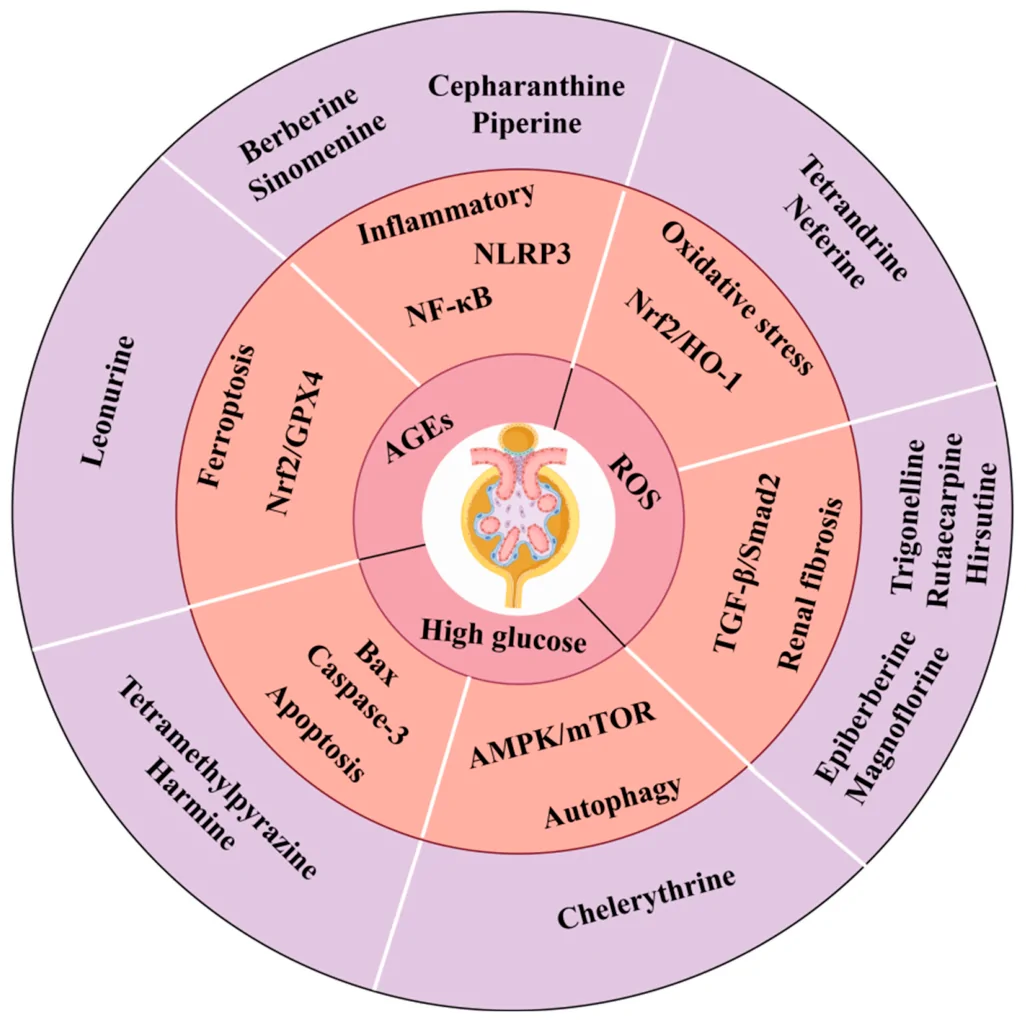
The renal protective mechanisms of alkaloid compounds in the treatment of diabetic nephropathy. Hyperglycemic stimulation leads to the accumulation of advanced glycation end products (AGEs) and the excessive production of reactive oxygen species (ROS), which activate several pathways: the NF-κB/NLRP3 inflammatory pathway, the Nrf2/HO-1 oxidative stress pathway, the TGF-β/Smad2 renal fibrosis pathway, the AMPK/mTOR autophagy pathway, the Bax/Caspase-3 apoptosis pathway, and the GPX4-mediated ferroptosis pathway. Various alkaloids can specifically regulate these pathways, thereby mitigating kidney damage associated with diabetic nephropathy. Created with Figdraw (by https://www.figdraw.com).

Alkaloid compounds, characterized by their diverse skeletal structures and the flexibility of their substituent modifications, can exert a range of effects, including anti-inflammatory, anti-oxidative, anti-fibrotic, anti-autophagic, anti-apoptotic, and anti-ferroptotic actions across multiple pathological processes. This diversity allows for targeted interventions in various pathogenic pathways. Among them, berberine and sinomenine inhibit the release of pro-inflammatory cytokines by blocking the TLR4/NF-κB pathway, whereas cepharanthine and piperine target the assembly of the NLRP3 inflammasome and the execution of pyroptosis, thereby truncating the inflammatory amplification loop at distinct nodes. Tetrandrine and neferine enhance the endogenous antioxidant enzyme system by activating the Nrf2/ARE transcriptional program. Epiberberine, trigonelline, rutaecarpine, and hirsutine suppress ECM deposition and EMT progression by interfering with the TGF-β/Smad pathway. Chelerythrine restores autophagic flux through the AMPK/mTOR axis. Tetramethylpyrazine and harmine protect the survival of intrinsic renal cells by regulating the apoptosis pathway. Leonurine, as a GPX4 regulator, for the first time integrates the ferroptosis mechanism into the antidiabetic nephropathy effects of alkaloids. These findings suggest that alkaloids do not act through a single pathway but rather exert their renoprotective effects synergistically by simultaneously modulating multiple pathogenic nodes. This intervention strategy for DN, a complex, multifactorial disease, aligns with its pathological essence (see Figure 9).

This review also acknowledges that the existing literature on alkaloid compounds is unevenly represented. While compounds such as berberine and tetrandrine have been substantiated by numerous independent studies, sinomenine, hirsutine, and trigonelline have also demonstrated relatively robust, multi-pathway evidence following supplementary searches. However, certain compounds, such as chelerythrine, have only been reported in a limited number of independent studies to date. Despite our efforts to broaden the scope of our search, the number of original studies that fulfill the inclusion criteria for this paper remains constrained. Consequently, for compounds with a weak evidence base, the conclusions should be interpreted as preliminary findings that necessitate validation through further rigorous in vivo and in vitro studies.

It is important to emphasize that the pharmacological effects of alkaloids extend beyond the local pathological regulation of the kidneys. Many alkaloids exhibit a dual mode of action, integrating upstream etiological prevention with downstream local renal intervention. Berberine, tetramethylpyrazine, and piperine serve as examples of these alkaloids, which can directly target pathological processes such as renal inflammation, oxidative stress, and fibrosis, thereby mitigating downstream damage associated with DN. Additionally, they can rectify the systemic pathological state of diabetes by enhancing glucose and lipid metabolism, reshaping the gut microbiota, protecting the systemic vascular endothelium, and regulating epigenetic mechanisms. This comprehensive approach reduces the risk of DN at its source [24,25,26]. Therefore, when assessing the therapeutic potential of alkaloids for DN, it is crucial to consider their systemic metabolic regulatory effects alongside their local renal protective effects, rather than concentrating solely on downstream interventions at the renal level.

Despite substantial preclinical evidence, the translation of alkaloid compounds into clinical treatments for DN continues to encounter several significant challenges. Firstly, the majority of in vivo activity data are derived from rodent models, particularly STZ-induced models, which exhibit considerable differences from human diabetic nephropathy regarding disease progression, metabolic context, and the degree of fibrosis [109]. Although db/db mice provide a closer approximation of type 2 diabetes, their homogeneous genetic background fails to fully encapsulate the heterogeneity observed in human DN [110]. Moving forward, it is imperative to utilize human-derived organoids, conditional knockout mice, and precision animal models informed by multi-omics approaches to improve the predictive validity of preclinical evaluations. Secondly, insufficient in vivo exposure presents a critical barrier to the efficacy of alkaloids. Quaternary ammonium alkaloids, such as berberine, are characterized by low oral bioavailability. The enterohepatic circulation and the activity of P-glycoprotein further contribute to significant interindividual variability [111,112]. Recently, notable advancements have been made in the field of nanodelivery systems, particularly with the development of modified nanoparticles based on chitosan, which have markedly improved the oral absorption of alkaloids. In a rat model of diabetic nephropathy, this nanocomposite formulation increased the oral bioavailability of berberine by 4.4-fold compared to the free drug and significantly enhanced its efficacy in ameliorating renal function and renal tissue pathology [113]. Finally, the identification of key pharmacologically active groups based on SAR studies and their targeted modification is a fundamental strategy for optimizing the pharmacokinetic properties of alkaloid lead compounds while minimizing their toxicity [114]. The primary aim of structural modification is to enhance pharmacokinetic properties by increasing oral absorption, prolonging half-life, and reducing clearance. This approach consequently leads to improved bioavailability and a decrease in dosing frequency [115]. Due to the diversity of their skeletal structures and the flexibility of their substituent modifications, alkaloid compounds can exert a range of effects, including anti-inflammatory, anti-oxidative, anti-fibrotic, anti-autophagic, anti-apoptotic, and anti-ferroptotic actions in various pathological processes. This versatility enables targeted interventions in different pathogenic pathways [116,117]. Alkylation at the C-9 and C-13 positions of berberine can reduce the positive charge on the quaternary ammonium nitrogen, thereby weakening P-glycoprotein efflux and improving lipophilicity [118]. Additionally, ring-opening or heteroatom substitution at the benzodioxol ring of piperine can enhance binding selectivity for the NLRP3 target [119]. Introducing a halogen or ester side chain into the phenolic hydroxyl group at the C-1 position of sinomenine can enhance NF-κB inhibitory activity and improve metabolic stability [120]. Furthermore, combination therapy shows promise; for instance, piperine, a dual inhibitor of CYP3A4 and P-glycoprotein, can increase the in vivo exposure of berberine, thereby achieving synergistic renal protection.

In summary, the potential of alkaloids in the treatment of DN is evident. The transformation of natural products into effective drugs for combating DN relies on multidisciplinary collaboration and the guidance of clinical research aligned with the principles of precision medicine, which is anticipated to overcome research and development (R&D) barriers. The ultimate benefit of this endeavor lies in the enhancement of patient care.

## Figures and Tables

**Table 1:** (**A**) Clinical studies of berberine—study design and basic information. (**B**) Clinical studies of berberine—endpoints and statistical outcomes.

(**A**)					
**Clinical Trials.gov Identifier**	**Study Title**	**Phases**	**Sample Sizes**	**Follow-Up Duration**	**Current Status**
NCT02808351	Berberine Prevent Contrast-induced Nephropathy in Patients with Diabetes	Phases IV	800	72 h	Completed (December 2021)
NCT00462046	Efficacy and Safety of Berberine in the Treatment of Diabetes with Dyslipidemia	Phases III	120	3 months	Completed (September 2006)
NCT03029390	Effect of Berberine Versus Metformin on Glycemic Control, Insulin Sensitivity and Insulin Secretion in Prediabetes	Phases IV	28	Baseline to week 14	Active, not recruiting
NCT00425009	Therapeutic Effects of Berberine in Patients with Type 2 Diabetes	Phases II	70 (36 reported)	13 weeks	Completed (December 2004)
NCT04698330	Effects Of Berberine Plus Inulin on Diabetes Care in Patients With LADA	Phases IV	240	1 year after start of trial	Not yet recruiting
NCT05749874	Effects of Berberine on Preventing Cardiovascular Disease and Diabetes Mellitus	Phases IV	2024	Intervention Period: 1 year	Active, not recruiting
(**B**)					
**Clinical Trials.gov Identifier**	**Primary Endpoints**		**Statistical Significance (If Reported)**		
NCT02808351	An absolute increase in serum creatinine ≥ 0.5 mg/dL (≥44.2 μM) or ≥25% increase in SCr from baseline to 72 h after the procedure		Not posted on registry; no linked publication found in PubMed.		
NCT00462046	FBG, 2h-PG, HbA1c, serum TG, TC, HDL-C, LDL-C		FBG (*p* < 0.0001); 2h-PG (*p* < 0.0001); HbA1c (*p* < 0.0001); TG (*p* < 0.0001); TC (*p* < 0.0001); LDL-C (*p* < 0.0001); HDL-C (*p* = 0.002)		
NCT03029390	FBG (baseline to week 14)		No results available (trial active, not recruiting; no publication identified).		
NCT00425009	HbA1c at week 13		HbA1c (*p* < 0.01)		
NCT04698330	Change in HbA1c		No results available (estimated completion: December 2026)		
NCT05749874	Glucose metabolism status at 1 year post-randomization		No results available (estimated completion: December 2026)		

Note: For NCT00425009, the 70 cases listed on the registration platform represent the planned screening target. However, only 36 subjects were actually enrolled and included in the analysis due to screening failures and restrictions on the inclusion criteria. The data reported in this table are based on the published results. Abbreviations: SCr, serum creatinine; FBG, fasting blood glucose; 2h-PG, 2 h plasma glucose; HbA1c, glycated hemoglobin A1c; TG, triglycerides; TC, total cholesterol; HDL-C, high-density lipoprotein cholesterol; LDL-C, low-density lipoprotein cholesterol.

**Table 2:** Studies on the mechanisms of action of alkaloids against diabetic nephropathy.

Compound	Source	Categories	Experiment Type	Model/Cell Line	STZ/HG Induction Protocol	Treatment Protocol	Major Effects	Ref.
Berberine	*Phellodendron amurense* Rupr.	Quaternary ammonium isoquinoline alkaloids	In vivo	Male SD rats (250 ± 20 g)	STZ 65 mg/kg, i.p., single injection	50, 100, 200 mg/kg/d, i.g., 8 weeks	↓: IL-1β, IL-6, MCP-1, TLR4, p-IκBα, p-p65	[27]
			In vitro	Podocytes	HG 30 mM, treatment 24 h	30 μM, co-treatment 24 h		
			In vitro	MCs	LPS 10 nM, treatment 30 min and 12 h	10, 30, 90 μM, pretreatment 24 h	↓: IκB-α, ICAM-1, TGF-β1, iNOS, FN	[28]
Sinomenine	*Sinomenium acutum* (Thunb.) Rehd. et Wils.	Morphinan-type isoquinoline alkaloid	In vivo	Male db/db mice (40 ± 6 g), 12 weeks old	Spontaneous type 2 diabetic model	62.4 mg/kg/d, i.g., 8 weeks	↓: IL-1β, IL-6, TNF-α, MCP-1, cGAS, STING, TBK1, IRF3, p-TBK1, p-IRF3, NF-κB	[29]
			In vitro	HrGECs	HG 30 mM, treatment 24 h	50, 100 μM, co-treatment 24 h	↓: IL-1β, IL-18	[30]
Cepharanthine	*Stephania japonica* (Thunb.) Miers	Bisbenzylisoquinoline alkaloids	In vivo	Male SD rats (180–220 g)	STZ 50 mg/kg, i.p., single injection	10 mg/kg/d, i.p., 8 weeks	↓: p-ERK, p-p38MAPK, p-JNK, TNF-α, IL-1β, TXNIP, NLRP3	[31]
Piperine	*Piper nigrum*	Piperidine alkaloids				30 mg/kg/d, i.p., 8 weeks		
Tetrandrine	*Stephania tetrandra* S. Moore	Bisbenzylisoquinoline alkaloids	In vivo	Wistar rats (100–200 g), 6–8 weeks old	STZ 50 mg/kg, i.p., single injection	5, 15, 30 mg/kg/d, i.g., 8 weeks	↑: SOD, p-Nrf2/Nrf2, HO-1 ↓: MDA, LDH, ROS, TNF-α	[32]
Neferine	*Nelumbo nucifera*	Bisbenzylisoquinoline alkaloids	In vivo	Male C57BL/6 mice, 6 weeks old	HFD 1 month + STZ 100 mg/kg, i.p., single injection	60, 120, 240 mg/kg/d, i.g., 12 weeks	↑: SOD, GSH-Px, Nrf2, HO-1 ↓: MDA	[33]
			In vitro	HMCs	HG 25 mM, treatment 48 h	1600.5 μM (≈1 mg/mL, MW 624.8), co-treatment 48 h		
Epiberberine	*Coptis chinensis* Franch.	Quaternary ammonium isoquinoline alkaloid	In vivo	Male db/db mice, 7 weeks old	Spontaneous type 2 diabetic model	50, 100, 200 mg/kg/d, i.g., 8 weeks	↓: Agt, TGF-β1, Smad2, Col-IV	[34]
			In vitro	SV40 MES 13	HG 30 mM, treatment 24 h	20, 40, 80 μM, co-treatment 24 h	↓: TGF-β1, Smad2	
Magnoflorine	*Magnolia biondii* Pamp.	Benzylisoquinoline Alkaloids	In vivo	Male SD rats, 6–8 weeks old	STZ 70 mg/kg, i.p., single injection	100 mg/kg/d, i.g., 30 days	↓: FN, α-SMA, Col-IV TGF-β1, p-Smad2/3	[35]
			In vitro	SV40 MES 13	HG 25 mM, treatment 48 h	292 μM (≈100 μg/mL, MW 342), co-treatment 48 h	↓: FN, α-SMA, Col-IV, TGF-β1	
Trigonelline	*Trigonella foenum-graecum* L.	Pyridine alkaloids	In vivo	Male db/db mice, 8 weeks old	Spontaneous type 2 diabetic model	50, 100 mg/kg/d, i.g., 8 weeks	↑: Smad7 ↓: FN, α-SMA, Col-III, Col-IV, p-Smad2, p-Smad3, TGFβRI	[36]
			In vitro	HK-2	HG 50 mM, treatment 36 h	80 μM, pretreatment 36 h	↑: Smad7, E-cadherin ↓: Vimentin, TGFβRI, p-Smad2, p-Smad3	
			In vitro	HMCs	HG 30 mM, treatment 48 h	50, 100, 200 μM, co-treatment 24 h	↓: FN, Col-IV, Wnt4, Wnt5a, β-catenin, TCF4, Cyclin D1, CDK4	[37]
Rutaecarpine	*Euodia rutaecarpa* (Juss.) Benth.	Quinazoline alkaloids	In vivo	Male db/db mice, 6–8 weeks old	Spontaneous type 2 diabetic model	5, 10, 20 mg/kg/d, i.g., 12 weeks	↓: FN, Col-IV, α-SMA, TGF-β1, p-Smad3	[38]
			In vitro	GMCs	HG 30 mM, treatment 24 h	0.5, 1, 2 μM, co-treatment 24 h	↓: FN, Col-IV, α-SMA, TGF-β1, p-Smad3	
Hirsutine	*Uncaria rhynchophylla* (Miq.) Miq. ex Havil.	Monoterpenoid indole alkaloids	In vivo	Male db/db mice, 6 weeks old	Spontaneous type 2 diabetic model	20 mg/kg/d, i.g., 8 weeks	↑: Smad7 ↓: TGF-β1, p-Smad2, p-Smad3	[39]
Chelerythrine	*Chelidonium majus* L.	Benzylisoquinoline alkaloids	In vivo	Male SD rats (180 ± 20 g), 8–10 weeks old	STZ 55 mg/kg, i.p., single injection	10, 20 mg/kg/d, i.g., 42 days	↑: LC3-II, Beclin-1, ATG5, p-AMPK, p-ACC ↓: p62, p-mTOR	[40]
Tetramethylpyrazine	*Ligusticum sinense Chuanxiong*	Pyrazine alkaloids	In vivo	Male Wistar rats (180–200 g), 5 weeks old	Nicotinamide 210 mg/kg, i.p. + STZ 55 mg/kg, i.p. (15 min later)	100, 150, 200 mg/kg/d, i.g., 8 weeks	↓: p-Akt/Akt, Bcl-2 ↓: Bax, p-GSK-3β/GSK-3β, Cleaved caspase-3	[41]
Harmine	*Peganum harmala* L.	β-carboline-type indole alkaloids	In vivo	Male Wistar rats	STZ 60 mg/kg, i.p., single injection	10 mg/kg/d, i.p., 2 weeks	↑: Bcl-2, p-AMPK ↓: p-ATM, p-p53, Bax, Caspase-3	[42]
Leonurine	*Leonurus japonicus* Houtt.	Phenolic acid-type organic amine alkaloids	In vivo	Male C57BL/6J mice (18–20 g), 4 weeks old	HFD 2 weeks + STZ 50 mg/kg, i.p., continuous 5 days	60 mg/kg/d, i.g., 12 weeks	↑: GPX4, FTL, FTH, GSH ↓: MDA	[43]
			In vitro	HUVEC	HG 30 mM, treatment 24 h	40 μM, co-treatment 24 h	↑: GPX4, FTH, FTL	

Note: Arrow symbols indicate significant changes in the indicators after drug administration: an upward arrow (↑) denotes upregulation or enhanced activity; a downward arrow (↓) denotes downregulation or reduced activity. Abbreviations: MCs, mesangial cells; HrGECs, human renal glomerular endothelial cells; HMCs, human mesangial cells; SV40 MES 13, mouse glomerular mesangial cells; HK-2, human renal proximal tubular epithelial cells; GMCs, glomerular mesangial cells; HUVEC, human umbilical vein endothelial cells; STZ, streptozotocin; HG, high glucose; LPS, lipopolysaccharide; HFD, high-fat diet; d, day; h, hour; i.g., intragastric administration; i.p., intraperitoneal injection; MW, molecular weight; IL-1β, interleukin-1 beta; IL-6, interleukin-6; MCP-1, monocyte chemoattractant protein-1; TLR4, toll-like receptor 4; p-IκBα, phosphorylated inhibitor of nuclear factor kappa b alpha; p-p65, phosphorylated nuclear factor kappa b p65; IκB-α, inhibitor of nuclear factor kappa b alpha; ICAM-1, intercellular adhesion molecule 1; TGF-β1, transforming growth factor beta 1; iNOS, inducible nitric oxide synthase; FN, fibronectin; TNF-α, tumor necrosis factor alpha; cGAS, cyclic gmp-amp synthase; STING, stimulator of interferon genes; TBK1, tank-binding kinase 1; IRF3, interferon regulatory factor 3; p-TBK1, phosphorylated tank-binding kinase 1; p-IRF3, phosphorylated interferon regulatory factor 3; NF-κB, nuclear factor kappa b; IL-18, interleukin-18; p-ERK, phosphorylated extracellular signal-regulated kinase; p-p38MAPK, phosphorylated p38 mitogen-activated protein kinase; p-JNK, phosphorylated c-jun n-terminal kinase; TXNIP, thioredoxin-interacting protein; NLRP3, nod-like receptor family pyrin domain containing 3; SOD, superoxide dismutase; p-Nrf2, phosphorylated nuclear factor erythroid 2-related factor 2; Nrf2, nuclear factor erythroid 2-related factor 2; HO-1, heme oxygenase-1; MDA, malondialdehyde; LDH, lactate dehydrogenase; ROS, reactive oxygen species; GSH-Px, glutathione peroxidase; Nrf2, nuclear factor erythroid 2-related factor 2; Agt, angiotensinogen; Smad2, smad family member 2; Col-IV, collagen type IV; α-SMA, alpha-smooth muscle actin; p-Smad2/3, phosphorylated smad family member 2/3; Smad7, smad family member 7; Col-III, collagen type III; p-Smad2, phosphorylated smad family member 2; p-Smad3, phosphorylated smad family member 3; TGFβRI, transforming growth factor beta receptor I; E-cadherin, epithelial cadherin; Wnt4, wingless-type mmtv integration site family member 4; Wnt5a, wingless-type mmtv integration site family member 5a; β-catenin, beta-catenin; TCF4, transcription factor 4; Cyclin D1, cyclin d1; CDK4, cyclin-dependent kinase 4; LC3-II, microtubule-associated protein 1 light chain 3 beta-II; Beclin-1, beclin-1; ATG5, autophagy related 5; p-AMPK, phosphorylated amp-activated protein kinase; p-ACC, phosphorylated acetyl-coa carboxylase; p62, sequestosome 1 (p62); p-mTOR, phosphorylated mammalian target of rapamycin; p-Akt/Akt, phosphorylated akt/akt; Bcl-2, b-cell lymphoma 2; Bax, bcl-2-associated x protein; p-GSK-3β, phosphorylated glycogen synthase kinase 3 beta; GSK-3β, glycogen synthase kinase 3 beta; p-ATM, phosphorylated ataxia telangiectasia mutated; p-p53, phosphorylated tumor protein p53; GPX4, glutathione peroxidase 4; FTL, ferritin light chain; FTH, ferritin heavy chain; GSH, glutathione.

**Table 3:** Structure–activity relationships of alkaloids in diabetic nephropathy.

Chemical Class	Compounds	Structural Features	Effective Dose Range (In Vivo/In Vitro)	Pharmacological Action	Refs.
Quaternary ammonium isoquinolines	Berberine (Figure 1A), Epiberberine (Figure 1G)	Isoquinoline backbone; quaternary N^+^; methylenedioxy ring; methoxy groups	In vivo: 50–200 mg/kg; in vitro: 10–90 μM	Anti-inflammation (TLR4/NF-κB); anti-fibrosis (TGF-β1/Smad)	[27,28,34]
Bisbenzylisoquinolines	Cepharanthine (Figure 1C), Tetrandrine (Figure 1E), Neferine (Figure 1F)	Two isoquinoline units connected by ether bridges; multiple phenolic OH; methoxy/methylenedioxy	In vivo: 10 mg/kg (Ceph.), 5–30 mg/kg (Tet.), 60–240 mg/kg (Nef.); in vitro: 1 μg/mL (Nef.)	Antioxidant (Nrf2/HO-1); anti-inflammation (NLRP3)	[31,32,33]
Morphinan-type isoquinolines	Sinomenine (Figure 1B)	Morphinan skeleton; bridging ether; C-4 phenolic OH; C-6 ketone; C-7 methoxy	In vivo: 20–62.4 mg/kg; in vitro: 50–100 μM	Anti-inflammation (NF-κB; cGAS/STING/NF-κB)	[29,30]
Pyridine alkaloids	Trigonelline (Figure 1I)	N-methylpyridinium ring; C-3 carboxylate; zwitterionic	In vivo: 50–00 mg/kg; in vitro: 80 μM	Anti-fibrosis (upregulates Smad7; inhibits TGF-β1/Smad)	[36,37]
Piperidine alkaloids	Piperine (Figure 1D)	Piperidine amide; methylenedioxyphenyl ring; conjugated diene chain	In vivo: 30 mg/kg (combined with cepharanthine 10 mg/kg);	Anti-inflammation (NLRP3 inflammasome)	[31]
Quinazoline alkaloids	Rutaecarpine (Figure 1J)	Quinazolinone fused with indole; planar conjugated ring; N-14 methyl	In vivo: 5–20 mg/kg; in vitro: 0.5–2 μM	Anti-fibrosis (TGF-β1/Smad3)	[38]
Monoterpenoid indole alkaloids	Hirsutine (Figure 1K)	Tetracyclic monoterpenoid indole skeleton; indole N-H; C-17 methoxycarbonyl	In vivo: 20 mg/kg	Anti-fibrosis (TGF-β/Smad; upregulates Smad7)	[39]
Pyrazine alkaloids	Tetramethylpyrazine (Figure 1M)	Pyrazine ring; 2,3,5,6-tetramethyl symmetric substitution	In vivo: 100–200 mg/kg	Anti-apoptosis (PI3K/Akt/GSK-3β)	[41]
β-Carboline indole alkaloids	Harmine (Figure 1N)	β-Carboline tricyclic skeleton; free indole N-H; C-7 methoxy; C-1 methyl	In vivo: 10 mg/kg	Anti-apoptosis (AMPK activation; ATM-p53 inhibition)	[42]
Organic amine alkaloids	Leonurine (Figure 1O)	Guanidine group; phenolic acid ester; C-4 phenolic OH; C-3, C-5 dimethoxy	In vivo: 60 mg/kg; in vitro: 40 μM	Anti-ferroptosis (GPX4/GSH; Nrf2/GPX4)	[43]

Abbreviations: TLR4, toll-like receptor 4; NF-κB, nuclear factor kappa-B; NLRP3, NOD-like receptor protein 3; TGF-β1, transforming growth factor-β1; Smad, small mothers against decapentaplegic; Nrf2, nuclear factor erythroid 2-related factor 2; HO-1, heme oxygenase-1; cGAS, cyclic GMP-AMP synthase; STING, stimulator of interferon genes; GPX4, glutathione peroxidase 4; GSH, glutathione; PI3K, phosphoinositide 3-kinase; Akt, protein kinase B; GSK-3β, glycogen synthase kinase-3β; AMPK, AMP-activated protein kinase; ATM, ataxia telangiectasia mutated.

## Data Availability

Data sharing is not applicable to this article as no new data were created or analyzed in this study.

## Supplementary Materials

The following supporting information can be downloaded at https://www.mdpi.com/article/10.3390/ijms27198938/s1.

## Abbreviations

The following abbreviations are used in this manuscript:

TLR4	Toll-Like Receptor 4
NF-κB	Nuclear Factor-κB
NLRP3	NOD-Like Receptor Family Pyrin Domain Containing 3
MyD88	Myeloid Differentiation Primary Response 88
p65-p50	NF-κB p65-p50 heterodimer
MD-2	Myeloid differentiation protein-2
p-IκBα	Phosphorylated Inhibitor of Nuclear Factor κB Alpha
p-p65	Phosphorylated NF-κB p65 subunit
NF-κB p65	NF-κB p65 subunit
ICAM-1	Intercellular Adhesion Molecule 1
iNOS	Inducible Nitric Oxide Synthase
NF-κB p50	NF-κB p50 subunit
cGAS	Cyclic GMP-AMP Synthase
STING	Stimulator of Interferon Genes
TBK1	TANK-Binding Kinase 1
IRF3	Interferon Regulatory Factor 3
NACHT	NAIP, CIITA, HET-E, and TP1 homology domain
PYD	Pyrin domain
ASC	Apoptosis-associated speck-like protein containing a CARD
CARD	Caspase recruitment domain
ARIH2	Ariadne RBR E3 ubiquitin protein ligase 2
TXNIP	Thioredoxin-Interacting Protein
Cys151	Cysteine 151
Cys273	Cysteine 273
Cys288	Cysteine 288
sMaf	Small muscu loaponeurotic fibrosarcoma
p-Nrf2	Phosphorylated Nuclear Factor Erythroid 2-Related Factor 2
Smad2	SMAD family member 2
Smad3	SMAD family member 3
Smad7	SMAD family member 7
Smad4	SMAD family member 4
Agt	Angiotensinogen
KDM3A	Lysine-Specific Demethylase 3A
siRNA	Small Interfering RNA
MH2	MAD homology 2 domain
HK-2	Human kidney-2 (cell line)
CK2α	Protein Kinase CK2 Catalytic Subunit Alpha
p62/SQSTM1	Sequestosome 1
p-Akt	Phosphorylated Protein Kinase B
Akt	Protein Kinase B
p-GSK-3β	Phosphorylated Glycogen Synthase Kinase 3 Beta
GSK-3β	Glycogen Synthase Kinase 3 Beta
Bax	Bcl-2-associated X protein
p-ATM	Phosphorylated Ataxia Telangiectasia Mutated
ATM	Ataxia Telangiectasia Mutated
p-p53	Phosphorylated Tumor Protein p53
p53	Tumor Protein p53
Bcl-2	B-cell lymphoma 2
H_2_O_2_	Hydrogen Peroxide
