# Supplementary material for: Multi-Target Regulation of Diabetic Nephropathy by Alkaloids: Molecular Targets, Signaling Pathways and Therapeutic Potential
Source: Int J Mol Sci. 2026 Oct 8;27(19):8938. doi: 10.3390/ijms27198938 (PMC13665509; doi:10.3390/ijms27198938)
Supplement: Supplementary file 1 [file ijms-27-08938-s001.zip › Supplementary Note S1.pdf]

**Supplementary Note S1.** Detailed structure-activity relationship analysis of alkaloids in DN. This Supplementary Note corresponds to Section 5 of the main text. It provides a detailed SAR discussion for each chemical class of alkaloids.

**S5.1 Quaternary ammonium isoquinoline alkaloids-Berberine, Epiberberine**

Quaternary ammonium isoquinoline alkaloids, such as berberine and epiberberine, exhibit common structural features, including a protoberberine core, a quaternary ammonium positive nitrogen atom, a methoxy group, and a methylenedioxy ring. Among these features, the quaternary ammonium nitrogen atom serves as the key structural basis for their biological activity, facilitating electrostatic interactions with negatively charged amino acid residues on target proteins. The methylenedioxy ring contributes to maintaining the molecular planar conformation, thereby facilitating the embedding of the small molecule into protein pockets for binding. Berberine and epiberberine are positional isomers; their molecular formulas and core skeletons are identical, differing only in the arrangement of the methylenedioxy ring and the two methoxy groups on the aromatic skeleton. In berberine, the C-2 and C-3 positions of the A ring are occupied by a methylenedioxy ring, while the C-9 and C-10 positions of the D ring are occupied by dimethoxy groups; in epiberberine, this arrangement is reversed. This alteration in substituent position does not disrupt the planar conjugated skeleton of the molecule but reshapes the spatial distribution of hydrogen-bonding sites and hydrophobic regions on the molecular surface, thereby modifying the spatial matching pattern between the molecule and the binding pockets of different target proteins.The structure-activity relationship analysis indicates that the TLR4/NF-κB-associated protein pocket is both deep and open, characterized by a hydrophobic groove on its outer side. The methylenedioxy ring of berberine A potentially embeds into this outer hydrophobic region, while the dimethoxy group of the D-ring fits into the interior of the pocket, thereby disrupting TLR4 dimerization and inhibiting NF-κB nuclear translocation, which results in an anti-inflammatory effect. In contrast, the TGFβRI kinase ATP pocket exhibits a deep, narrow topological structure with a loose entrance, containing negatively charged residues and hydrogen bond donors within the groove. The rigid

methoxy ring of the D-ring in berberine can penetrate deep into the pocket with minimal hindrance, stabilizing the drug-receptor complex through hydrophobic interactions, hydrogen bonding, and the synergistic electrostatic effects of the quaternary ammonium positive nitrogen. Furthermore, the dimethoxy groups on the A ring face the open pocket entrance to facilitate binding, thereby enhancing its affinity for TGFβRI and inhibiting TGF-β1-induced Smad2/3 phosphorylation, which blocks the fibrotic signaling pathway.

### S5.2 Benzylisoquinoline Alkaloids

#### S5.2.1 Typical benzylisoquinoline alkaloid-Magnoflorine

Magnoflorine is a typical benzylisoquinoline alkaloid characterized by a fused benzyl-isoquinoline skeleton. This molecule features a quaternary ammonium cation structure, and its highly planar aromatic core provides an essential spatial framework for recognizing fibrosis-related target proteins. Unlike the dibenzyl dimer, magnoflorine exists as a monomeric benzylisoquinoline that possesses multiple free phenolic hydroxyl and methoxy groups on its core ring. The quaternary ammonium group imparts a stable positive charge, facilitating binding to negatively charged regions of TGF-β/Smad pathway proteins through electrostatic interactions. The phenolic hydroxyl groups act as hydrogen bond donors, establishing a hydrogen bond network with polar amino acid residues on TGF-β receptors and Smad2/3 proteins, thereby disrupting the assembly of receptor-Smad protein complexes. Additionally, these groups scavenge reactive oxygen species in the fibrotic microenvironment. The methoxy groups, functioning as hydrophobic moieties, interact with the phenolic hydroxyl groups, modulating the overall polarity and solubility of the molecule. The structure-activity relationship analysis indicates that the pharmacological activity of Magnoflorine in inhibiting the TGF-β/Smad fibrosis signaling pathway arises from the synergistic effects of its benzylisoquinoline monomeric core, the positively charged quaternary ammonium, and the polyphenolic hydroxyl groups. Consequently, the benzylisoquinoline monomeric skeleton serves as the structural foundation, while the quaternary ammonium cation, along with multiple phenolic hydroxyl and methoxy groups, constitutes the core pharmacologically active unit. Collectively, these

components facilitate Magnoflorine's ability to inhibit TGF-β/Smad signaling and reduce extracellular matrix deposition in the kidney, thereby demonstrating its antifibrotic effects.

**S5.2.2 Benzo[b]pyridine alkaloids among benzylisoquinoline alkaloids-Chelerythrine**

Chelerythrine is a benzo[b]pyridine alkaloid that belongs to the benzylisoquinoline class. Its core structure consists of a planar benzo[b]pyridine fused aromatic ring system. The molecule features a quaternary ammonium nitrogen atom, a methoxy group, and a methylenedioxy ring. Its highly conjugated, planar aromatic core allows it to embed itself into the hydrophobic pockets of proteins, serving as the structural basis for its recognition of target proteins. The benzo[b]pyridine core contains a quaternary ammonium cation, and this positive charge generates electrostatic attraction, facilitating targeted binding to negatively charged amino acid residues surrounding the catalytic domain of the AMPK protein. The fused, planar aromatic structure enhances hydrophobic interactions, promoting the phosphorylation and activation of AMPK, which subsequently inhibits downstream mTOR signaling. Structure-activity relationship analyses indicate that the pharmacological property of chelerythrine in activating AMPK-dependent autophagy may arise from its planar benzo[b]pyridine fused ring system and quaternary ammonium cation pharmacophore. In summary, the fused aromatic benzopyridine core provides the structural foundation, while the quaternary ammonium cation and the extensive, highly conjugated planar aromatic system function as the core pharmacophore, synergistically mediating the biological functions of chelerythrine in activating the AMPK-mTOR autophagy pathway and alleviating kidney injury.

**S5.2.3 Bisbenzylisoquinolines-Cepharanthine, tetrandrine, neferine**

Dibenzylisoquinoline alkaloids, including cepharanthine, tetrandrine, and neferine, exhibit a dimeric core skeleton formed by the linkage of two isoquinoline monomers through an aryl ether bridge (Ar-O-Ar). This dimeric structure leads to a substantial molecular volume and a flexible, tunable three-dimensional conformation, which serves as the structural basis for these compounds' interactions with various

target proteins. The molecular framework is characterized by multiple phenolic hydroxyl groups, methoxy groups, and methylenedioxy rings. The number of ether bridges, the sites of linkage between aromatic rings, and the substitution patterns of methoxy or methylenedioxy groups are crucial pharmacophores that differentiate the activity profiles of various subtypes. Cepharanthine and tetrandrine contain two aryl ether bridges and lack free phenolic hydroxyl groups, being predominantly substituted by methoxy and methylenedioxy groups. In contrast, neferine belongs to the dibenzyl tetrahydroisoquinoline subtype, where the two monomers are dimerized via a single diaryl oxygen bridge while retaining one free phenolic hydroxyl group. This oxygen bridge constrains the spatial orientation of the two isoquinoline units, resulting in a spatial conformation that fits the Nrf2 protein binding pocket. In Neferine, the free phenolic hydroxyl group functions as the primary pharmacologically active component, responsible for scavenging free radicals and exerting antioxidant effects. The adjacent methoxy group stabilizes the free radical intermediates generated by the phenolic hydroxyl group through its electron-donating properties, thereby promoting the dissociation of Keap-1 from Nrf2 and activating the downstream HO-1 antioxidant response. In summary, the dibenzylisoquinoline dimeric core serves as the structural foundation for this class of alkaloids. The aromatic ether bridge linkage, along with the arrangement of phenolic hydroxyl and methoxy substituents, constitutes the core pharmacological units. Variations in these substituents may allow different members of the family to mediate two distinct biological effects: the inhibition of inflammasomes or the activation of the Nrf2 antioxidant response.

### S5.3 Morphinan-type isoquinolines-Sinomenine

Sinomenine is a typical morphinan-type isoquinoline alkaloid characterized by a distinctive morphinan skeleton. This compound combines a rigid fused-ring structure with a unique bridged ether bond configuration. The unsaturated nature of the C-ring and the C-6-keto substitution are hallmark structural features that differentiate it from other isoquinoline alkaloids. The intact morphinan skeleton provides a structural foundation that facilitates stable binding to target proteins and underlies its pharmacological activity. Notably, the C-4 phenolic hydroxyl group in the sinomenine

molecular structure is likely the primary pharmacologically active group responsible for regulating NF-κB inflammatory signaling and mediating its anti-inflammatory protective effects. Structure-activity relationship analysis indicates that the biological effects of sinomenine in inhibiting inflammatory activation and blocking inflammatory cascades may depend on the structural integrity of its phenolic hydroxyl groups. Specifically, the free phenolic hydroxyl group on the benzene ring serves as a potent hydrogen-bond donor, establishing specific hydrogen bonds with amino acid residues of key proteins in the NF-κB pathway. This interaction effectively disrupts the NF-κB transactivation process and inhibits the transcriptional activation of inflammatory genes. Concurrently, the intact morphinan skeleton and the bridging ether bond structure preserve the stability of the molecular conformation, ensuring effective docking and binding of the small molecule to inflammatory targets. The ketone group at C6 and the methoxy group at C7 may facilitate weak hydrogen bonding interactions, further stabilizing the conformation of the drug-target complex. Additionally, the C-3 methoxy group contributes to the stability of the molecular skeleton and its overall physicochemical properties through its hydrophobic characteristics. In summary, the rigid morpholine-type core of sinomenine serves as the structural foundation for its biological activity, while the free phenolic hydroxyl group is the core pharmacologically active moiety responsible for its anti-inflammatory effects. These structural units work synergistically to mediate its targeted regulation of the NF-κB inflammatory pathway.

**S5.4 Pyridine alkaloids-Trigonelline**

Trigonelline is a typical pyridine alkaloid characterized by an N,N-dimethylpyridinium aromatic heterocyclic skeleton as its core. Its overall structure is highly stable and strongly planar, serving as the fundamental framework for recognizing and binding to target proteins. Unlike other alkaloids, trigonelline is attached to a carboxylate anion at the 3-position, endowing the molecule with the characteristics of a zwitterion, wherein a positive pyridinium charge coexists with a negative carboxylate charge. Structure-activity relationship analysis indicates that the

core pharmacological characteristics of trigonelline—specifically, the upregulation of Smad7 and the inhibition of the TGF-β1/Smad fibrosis signaling pathway—may arise from its zwitterionic structure. The positive charge of the N-methylpyridinium ion is capable of specifically binding to the negatively charged regulatory regions of the Smad7 gene and protein through electrostatic interactions, providing a fundamental driving force for the molecularly targeted anchoring of fibrosis-related targets. Additionally, the carboxylate group at the C3 position functions as a multi-hydrogen-bond receptor, establishing a stable hydrogen-bond network with polar amino acid residues of the target protein, which collectively enhances the expression and functional activation of Smad7. As a critical negative feedback regulator in the TGF-β1/Smad pathway, Smad7 effectively inhibits TGF-β1-mediated phosphorylation and nuclear translocation of Smad2/3, thereby preventing the excessive deposition of downstream fibrosis-related genes and extracellular matrix proteins. In summary, the pyridinium core of fenugreek alkaloid provides the structural foundation, while the amphiphilic charge distribution and the carboxylate group at the C3 position serve as the core pharmacological units, together mediating its biological functions of specifically activating Smad7 and blocking the TGF-β1/Smad fibrosis signaling pathway.

**S5.5 Piperidine alkaloids-Piperine**

Piperine is a typical piperidine alkaloid characterized by a saturated six-membered piperidine heterocycle. The molecule comprises a piperidinamide structure, an aromatic methylenedioxybenzene ring, and a long-chain conjugated unsaturated double-bond system, collectively forming a complete lipophilic planar aromatic conjugated system. This conjugated skeleton, along with its hydrophobic structure, may provide the structural basis for stable binding to inflammatory targets and the regulation of multiple inflammatory pathways. The piperine molecule possesses a hydrophobic backbone along with multiple hydrogen-bonding sites. Its rich structural units and clearly defined functional roles enable it to specifically target the NLRP3 inflammatory signaling axis. Structure-activity relationship analysis indicates that piperine's capacity to inhibit inflammasome activation and

inflammatory signal transduction may rely on the integrity of its "piperidinamide–methylenedioxy aromatic ring-conjugated alkene chain." In this configuration, the methylenedioxybenzene ring acts as the core hydrophobic pharmacologically active unit, allowing it to embed itself into the hydrophobic pocket of the NLRP3 pathway target through strong hydrophobic interactions. This mediates the initial recognition and binding between the molecule and the target protein. Additionally, the methoxy oxygen atom and the oxygen atom of the piperidamide bond function as hydrogen-bond acceptors, forming stable hydrogen bonds with polar residues of the target protein, which further stabilizes the drug's binding conformation. The long-chain conjugated double-bond system preserves the molecule's overall planar conjugated configuration, enhancing structural stability and membrane permeability. This ensures effective accumulation within cells and the exertion of its anti-inflammatory effects. In summary, the piperine heterocycle and the conjugated aromatic system constitute the foundation of the active structure, while the methylenedioxy and amide structures serve as key pharmacologically active groups, synergistically mediating its broad-spectrum inhibitory effects on multiple inflammatory pathways.

**S5.6 Quinazoline alkaloids-Rutaecarpine**

Rutaecarpine is a representative quinazoline alkaloid characterized by its core structure, which consists of a polycyclic aromatic fused ring system formed through the condensation of a quinazolinone ring and an indole ring. This compound exhibits a large, planar conjugated structure akin to that of β-carboline alkaloids, demonstrating significant rigidity and structural stability. A notable feature of the molecule is the methyl substitution at the 14-N position; this alkyl substituent plays a crucial role in precisely regulating the molecule’s lipophilicity, steric hindrance, and target-binding affinity. The analysis of structure-activity relationships indicates that rutaecarpine's capacity to inhibit extracellular matrix (ECM) deposition and block the TGF-β1/Smad3 fibrotic signaling pathway may rely on the integrity of its polycyclic skeleton and the 14-N-methyl substitution pattern. Specifically, the planar conjugated polycyclic skeleton can embed itself into the aromatic hydrophobic pockets of target

proteins associated with the TGF-β/Smad pathway through π-π stacking and hydrophobic interactions, providing a structural basis for the molecule's specific recognition of fibrosis targets. If the integrity of the polycyclic conjugated system is disrupted, resulting in a loss of planarity, the molecule will lose its capacity for targeted binding. Although the 14-N-methyl group does not directly engage in hydrogen bonding or electrostatic interactions, it significantly enhances the molecule's lipophilicity and cell membrane permeability, optimizing its pharmacological accumulation within cells while moderately regulating steric hindrance to ensure optimal spatial alignment between the small molecule and the target protein pocket. The synergistic action of these structural units inhibits TGF-β1-induced phosphorylation and activation of Smad3, blocking Smad3 nuclear translocation and the abnormal deposition of extracellular matrix components, such as collagen and fibronectin, downstream, thereby significantly delaying the progression of renal fibrosis. In conclusion, the indole-fused aromatic core of rutaecarpine (quinazoline) serves as the foundation of its activity, while the planar conjugated structure and the methyl substituent at the N14 position act as key functional units, collectively mediating its inhibitory effect on the TGF-β1/Smad3 fibrosis pathway.

**S5.7 Monoterpenoid indole alkaloids-Hirsutine**

The core structural feature of the monoterpene indole alkaloid hirsutine is its tetracyclic monoterpene indole skeleton, which is formed by the condensation of an indole ring and a quinolizidine ring system. This system comprises a six-membered piperidine ring at position C and a six-membered piperidone ring at position D, connected through a helical fusion. The N-H group on the indole ring is crucial for its biological activity; it can act as a hydrogen bond donor, facilitating interactions with target proteins. Meanwhile, the methoxycarbonyl group (-$COOCH_3$) at the C-17 position serves as a hydrogen bond acceptor, contributing to conformational stability. Structure-activity relationship analysis indicates that hirsutine's ability to regulate the TGF-β/Smad pathway and exert antifibrotic effects may be closely linked to its structural model characterized by a "rigid fused ring skeleton, flexible side chains, and specific chiral configuration." The indole N-H group functions as a hydrogen

bond donor, forming stable interactions with Smad7-related regulatory regions or TGF-β receptor complexes. In parallel, the methoxycarbonyl group at position C-17 synergistically stabilizes the conformation of the drug-target complex through hydrogen bonding and hydrophobic interactions. The combined action of these structural elements enables hirsutine to inhibit the specific recognition and phosphorylation of Smad2/3 by TGF-β1 through the upregulation of Smad7, thereby obstructing the TGF-β/Smads signaling pathway and reducing extracellular matrix deposition and renal fibrosis.

**S5.8 Pyrazine alkaloids-Tetramethylpyrazine**

The core structural feature of the pyrazine alkaloid tetramethylpyrazine is the highly symmetrical substitution of four methyl groups at positions 2, 3, 5, and 6 on the pyrazine ring. This ring constitutes the primary pharmacophore, and the lone pair of electrons on the nitrogen atom of the parent nucleus is essential for maintaining its biological activity. The four methyl groups confer appropriate lipophilicity to the molecule, facilitating its passage across the cell membrane and promoting intracellular accumulation. This mechanism enables the drug to achieve effective concentrations within target cells and interact with relevant signaling proteins. Structure-activity relationship analyses suggest that tetramethylpyrazine's capacity to regulate the PI3K/Akt/GSK-3β apoptotic pathway and protect renal juxtaglomerular cells may be closely associated with this symmetrical tetramethyl substitution pattern. The moderate lipophilicity provided by the methyl groups enhances transmembrane transport and intracellular accumulation, thereby allowing the drug to attain the effective intracellular concentration necessary for pathway regulation. Concurrently, the hydrophobic and electron-donating effects of the methyl groups contribute to the stabilization of its interaction with the ATP-binding pockets of PI3K/Akt/GSK-3β-related kinases. This interaction activates Akt, inhibits GSK-3β, upregulates Bcl-2, and downregulates Bax and cleaved caspase-3, ultimately resulting in a reduction of apoptosis in renal juxtaglomerular cells.

**S5.9 β-Carboline indole alkaloids-Harmine**

Harmine is a representative β-carboline-type indole alkaloid. Its core structure

comprises a fully aromatic, conjugated β-carboline tricyclic skeleton formed by the condensation of an indole ring and a pyridine ring; this planar conjugated structure underlies its biological activity. This compound exhibits well-defined structural features: the indole ring retains a free N-H group, while the benzene ring is substituted with a methoxy group at the C7 position and the pyridine ring is substituted with a methyl group at the C1 position. These structural units work synergistically to confer its unique biological functions. Structure-activity relationship analysis indicates that the core pharmacological units responsible for Harmine’s activation of the AMPK pathway and inhibition of the ATM-p53 apoptotic signaling pathway are the free N-H group on the indole ring and the methoxy group at the C7 position. The free N-H group on the indole ring functions as a hydrogen bond donor, specifically binding to target proteins; it is crucial for initiating AMPK activation and inhibiting the p53 apoptotic signaling pathways. Substitution modifications at this site, such as methylation or acetylation, directly lead to the loss of its core biological activity. Meanwhile, the methoxy group at C7 of the indole ring serves as a hydrogen-bond acceptor, facilitating the interaction between the molecule and the hydrophobic pocket of the target protein; the absence of this methoxy group significantly diminishes its pathway-regulating activity. Furthermore, the intact β-carboline aromatic conjugated skeleton is essential for maintaining its biological activity; hydrogenation of the pyridine ring disrupts the molecular conjugated system, markedly reducing pharmacological efficacy. Although the methyl group at C1 of the pyridine ring does not exert a direct pharmacological effect, it enhances the molecule’s lipophilicity, improves cellular permeability, and contributes to amplifying the overall biological effect. In summary, the β-carboline core of Harmine serves as the foundation for its activity, while the free N-H group on the indole ring and the methoxy group at C7 represent key active groups, suggesting that they may collaboratively mediate the regulation of the AMPK and ATM/p53 apoptotic signaling pathways.

**S5.10 Organic amine alkaloids-Leonurine**

Leonurine is classified as an organic amine alkaloid, characterized by a nitrogen

atom within an open-chain guanidinium structure, which notably lacks a nitrogen-containing heterocyclic core. Its molecular backbone comprises an ester bond that links cinnamic acid (4-hydroxy-3,5-dimethoxybenzoic acid) to 4-guanidynobutanol. Structure-activity relationship analyses indicate that the side-chain guanidino group may serve as the principal pharmacologically active moiety responsible for Leonurine's activation of the GPX4/GSH antioxidant axis and the inhibition of ferrocytosis. This guanidino group potentially stabilizes the GPX4 protein and sustains GSH levels through electrostatic and hydrogen bonding interactions, thereby mitigating lipid peroxidation. It presents a unique structural template for alkaloid intervention in ferrocytosis associated with diabetic nephropathy. Additionally, the phenolic hydroxyl group at the C-4 position of the benzene ring can directly scavenge reactive oxygen species, thereby synergistically inhibiting oxidative stress; however, methylation of this group results in the loss of its antioxidant activity. The dimethoxy groups at the C-3 and C-5 positions modulate the electron density of the benzene ring and enhance membrane permeability. Furthermore, the 4-carbon alkyl side chain and the ester bond preserve the molecular spatial conformation, ensuring effective binding of the guanidinium group to the target site; once the ester bond is hydrolyzed, the molecule's activity is significantly diminished.

In summary, certain bioactive groups recur across different classes of alkaloids and perform essential functions. Quaternary ammonium groups are frequently involved in the inhibition of anti-inflammatory signaling, while free phenolic hydroxyl groups generally mediate antioxidant effects. Methylenedioxy rings contribute to maintaining a planar molecular conformation, thereby enhancing binding affinity for targets. Additionally, various fused ring and ether bridge frameworks provide a foundational structure that supports the overall spatial conformation of the molecule. However, it is important to note that current structure-activity relationship studies on DN-related alkaloids exhibit significant shortcomings. Inferences regarding structure-activity relationship are primarily derived from molecular docking, derivative studies, and correlations with pharmacological phenotypes. Targeted structural modification experiments on DN models remain relatively scarce, and

comprehensive $IC_{50}$ quantitative data are lacking. Consequently, the aforementioned SAR relationships necessitate further experimental validation.
